# Effectiveness and cost-effectiveness of a peer-delivered, relational, harm reduction intervention to improve mental health, quality of life, and related outcomes, for people experiencing homelessness and substance use problems: protocol for the ‘SHARPS’ cluster randomised controlled trial

**DOI:** 10.1186/s13063-025-09364-x

**Published:** 2025-12-13

**Authors:** Tessa Parkes, Hannah Carver, Jennifer Boyd, Seonaidh Cotton, Suzanne Breeman, David Cooper, Mark Forrest, Rebecca Foster, Jake Hawthorn, Kate Hunt, Mary Kilonzo, Catriona Matheson, Margaret Maxwell, Stewart W. Mercer, Bernie Pauly, Graham Scotland, Wez Steele, Harry Sumnall, Jason Wallace, Lisa Macaulay, Graeme MacLennan

**Affiliations:** 1 Salvation Army Centre for Addiction Services and Research, Faculty of Social Sciences, University of Stirling, Stirling, Scotland FK9 4LA UK; 2 Centre for Healthcare Randomised Trials, University of Aberdeen, Aberdeen, AB25 2ZD UK; 3 School of Applied Sciences, Edinburgh Napier University, Sighthill Campus, Edinburgh, EH11 4BN UK; 4 Institute of Social Marketing and Health, University of Stirling, Stirling, FK9 4LA UK; 5 Health Economics Research Unit, University of Aberdeen, Aberdeen, AB25 2ZD UK; 6 Centre for Healthcare and Community Research, Faculty of Health Sciences and Sport, University of Stirling, Stirling, FK9 4LA UK; 7 Usher Institute, University of Edinburgh, Edinburgh, EH16 4UX UK; 8 Canadian Institute for Substance Use Research, University of Victoria, Victoria, BC V8P 5C2 Canada; 9 Scottish Drugs Forum, Glasgow, G1 3LN UK; 10 School of Psychology, Liverpool John Moores University, Liverpool, L2 2QP UK

**Keywords:** Cluster randomised controlled trial, Peer support, Homelessness, Social care, Substance use, Drugs, Alcohol

## Abstract

**Background:**

Those experiencing homelessness and problem substance use find it challenging to access the healthcare and treatment they need. The Supporting Harm Reduction through Peer Support (SHARPS) feasibility study demonstrated that Peer Navigators can help these individuals to improve their service engagement, increase access to opioid substitution therapy, and lead to reductions in drug use and risky injection practices. Specifically, participants indicated that the lived experience of Peer Navigators was particularly helpful by enabling the development of trusting relationships. A cluster randomised controlled trial (cRCT) will now assess the effectiveness and cost-effectiveness of a Peer Navigator intervention with this population.

**Methods:**

A two-arm, pragmatic, cRCT will be conducted with embedded cost-effectiveness and mixed methods process evaluations. Individuals will be recruited who are as follows: over the age of 18 years; experiencing/at risk of homelessness and self-report problem substance use; and attending The Salvation Army (TSA) homelessness services across 20 included clusters (towns/cities). Each cluster will be randomised (1:1) to either the intervention or control arm using covariate-constrained allocation based on area-level characteristics. The target sample size is 550 participants in total. A co-produced peer-delivered harm reduction, relational intervention lasting 12 months will be delivered to those in the intervention arm. Usual care will be social care via TSA Support Workers delivered within homelessness services. The co-primary outcomes will be mental health and quality of life, with harmful substance use, risk taking behaviours, social functioning, physical health, social outcomes, housing status, therapeutic alliance/accessibility, service utilisation, and relational empathy chosen as secondary outcomes. Data collection points are baseline, 6 and 12 months, for all measures. The primary timepoint of interest is 12 months after baseline measurement. Economic outcomes will be incremental cost per quality-adjusted life year (QALY) and per year in full capability (YFC) gained with the intervention versus standard homelessness service care, inclusive of costs to the NHS, local government and criminal justice, and the third-sector host organisation. The EQ-5D-5L and ICECAP-A will be used to calculate QALYs and YFC respectively. We will also conduct a cost-consequence analysis.

**Discussion:**

The results of this trial will be used to inform whether the SHARPS intervention has a positive impact on those experiencing homelessness and problem substance use and if it is cost-effective to roll it out across social care services.

**Trial registration:**

ISRCTN11094645 (https://doi.org/10.1186/ISRCTN11094645, registered April 5, 2024).

**Supplementary Information:**

The online version contains supplementary material available at 10.1186/s13063-025-09364-x.

## Administrative information

Note: the numbers in curly brackets in this protocol refer to SPIRIT checklist item numbers. The order of the items has been modified to group similar items (see http://www.equator-network.org/reporting-guidelines/spirit-2013-statement-defining-standard-protocol-items-for-clinical-trials/).

**Table:** 

Title {1}	Assessing the impact of peer-led harm reduction on people experiencing homelessness and problem substance use – a cluster RCT: The SHARPS study.
Trial registration {2a and 2b}.	ISRCTN11094645, prospectively registered (date of registration, 05 April 2024).
Protocol version {3}	Version 6 (approved 10/07/2025).
Funding {4}	This project is funded by the NIHR Health Technology Assessment Programme: NIHR150358. Department of Health and Social Care for English Excess Treatment Costs and The Salvation Army for Scottish Excess Treatment Costs.
Author details {5a}	^1^ Salvation Army Centre for Addictions Services and Research, Faculty of Social Sciences, University of Stirling, Stirling, FK9 4LA, United Kingdom. ^2^ Centre for Healthcare Randomised Trials, University of Aberdeen, Aberdeen, AB25 2ZD, United Kingdom. ^3^ School of Applied Sciences, Edinburgh Napier University, Edinburgh, EH11 4BN, United Kingdom. ^4^ Institute for Social Marketing and Health, University of Stirling, Stirling, FK9 4LA, United Kingdom. ^5^ Health Economics Research Unit, University of Aberdeen, Aberdeen, AB25 2ZD, United Kingdom. ^6^ Centre for Healthcare and Community Research, Faculty of Health Sciences and Sport, University of Stirling, Stirling, FK9 4LA, United Kingdom. ^7^ Usher Institute, University of Edinburgh, Edinburgh, EH16 4UX, United Kingdom. ^8^ Canadian Institute for Substance Use Research, University of Victoria, Victoria, BC, V8P 5C2, Canada. ^9^ Scottish Drugs Forum, Glasgow, G1 3LN, United Kingdom. ^10^ School of Psychology, Liverpool John Moores University, Liverpool, L2 2QP, United Kingdom.
Name and contact information for the trial sponsor {5b}	University of Stirling Rachel Beaton, rachel.beaton@stir.ac.uk
Role of sponsor {5c}	The sponsor played no part in study design; and will play no part in the collection, management, analysis, and interpretation of data; writing of the report; and the decision to submit the report for publication.

## Introduction

### Background and rationale {6a}

People experiencing homelessness are faced with significant social and economic challenges and are more likely to experience problem substance use and severe mental health challenges, all of which compound their risk of acute and chronic health problems [1, 2]. In 2022/2023, approximately 53,111 people experienced homelessness in Scotland [3], and 271,000 in England [4]. The lives of those experiencing homelessness are permeated with insecurity, trauma, violence, and stigma. There is also a particularly high burden of mental health problems, with approximately 30% of those experiencing homelessness reporting a mental health problem in a study conducted in Scotland [5]. Additionally, experiencing homelessness directly impacts the risk of death: those experiencing homelessness have a sevenfold increased risk of death related to drug use when compared with the general population [6]. Problematic use of alcohol and drugs can often lead to, as well as be a way of coping with, homelessness [7], and the co-occurrence of poor mental and physical health and problem substance use, or tri-morbidity [8], is therefore common. Risk of death is compounded when homelessness intersects with other aspects of severe disadvantage such as imprisonment, substance use, sex work, and/or severe mental illness [9, 10]. Additionally, in England, the impact of austerity through cuts to local authorities between 2008 and 2015 have led to higher rates of deaths due to drugs, suicide, and alcohol in deprived communities [11]. Evidence also suggests that close to one in three deaths of those experiencing homelessness could have been prevented with appropriate and timely healthcare intervention [12].

For people experiencing homelessness, it can be difficult to access healthcare and treatment given societal stigma, negative attitudes held by staff, and inflexible services [13, 14]. Given the rigidity of primary care hours, those experiencing homelessness often rely on emergency healthcare services [15]. Of this population, those with mental health problems, including problem substance use, are more likely to miss healthcare appointments and, as a result, are eight times more likely to die prematurely compared to those who did attend their appointments [16]. Harm reduction, peer delivered, and psychologically informed environment (PIEs) approaches have shown considerable promise in supporting people experiencing homelessness and problem substance use. Briefly, harm reduction involves a non-judgmental response to substance use and aims to meet the needs of individuals by reducing the harms associated with substance use without requiring abstinence/cessation [13]. Examples of harm reduction approaches include, but are not limited to, the following: overdose awareness and intervention training and naloxone provision; supplies of sterile injecting equipment; drug consumption rooms; and non-abstinence-based housing [13].

Meaningful involvement of peers (people with experience of homelessness and/or problem substance use) is key to a harm reduction approach [17] and previous work has shown that peer support can *reduce* substance use and related harm [14, 18], and *improve* quality of life [14, 19, 20], mental health [20], social functioning [21], housing/homelessness status [14, 20], vocational outcomes [18, 20], treatment engagement/acceptability [21, 22], access to healthcare [23], engagement with overdose prevention activities [24], and retention during COVID-19 isolation [25]. Importantly, such involvement can also benefit the peers delivering services themselves [14, 26]. In the UK, the National Institute for Health and Care Excellence (NICE) has issued guidelines [27] that emphasise the following factors as important for the provision of health and social care for people experiencing homelessness: peer support, role modelling, supporting attendance at appointments and navigating services, formation of trusting relationships, and provision of advocacy to facilitate continued engagement with services for those experiencing homelessness.

A PIE approach has become increasingly popular in homelessness settings in the UK [13, 28]. A PIE approach uses an individual’s experiences, including of trauma and environmental factors, to understand how they think, feel, and behave, and this understanding is then used to design and deliver appropriate services [13, 28]. PIE approaches have five key areas: developing greater psychological awareness of the needs of individuals; valuing training and support for all staff, volunteers, and clients; promoting a culture of learning and enquiry, including in service evaluation and improvement; enabling ‘spaces of opportunity’ which seek to create effective service environments; and a focus on the rules, roles, and responsiveness of the service which focuses on managing and improving relationships [29]. There is some evidence that PIEs-informed services can *improve* mental health and well-being, housing, and behavioural outcomes; engagement with health, substance use, and other care services; and *reduce* involvement with criminal justice and emergency services [13, 30–33]. While this is encouraging, limited studies have adopted a PIE approach in the field of substance use, and very few studies have specifically investigated the impact of combining PIEs and peer support approaches [13, 31, 34].

The Supporting Harm Reduction through Peer Support (SHARPS) intervention was developed to address the above evidence gaps and bring together harm reduction, peer-delivered, and PIE approaches to address the need for new ways of working in social care environments for those experiencing homelessness and problem substance use. The SHARPS feasibility study (2018–2020) examined whether a peer-delivered relational intervention was acceptable, accessible, and feasible to deliver to people experiencing homelessness and problem substance use in third sector homelessness settings in Scotland and England [35]. It was a single-arm pre-post design. In this feasibility study, four Peer Navigators were employed to support individuals (*n* = 68 participants) for up to 12 months based in outreach services and hostels run by three third sector organisations in Scotland and England. Qualitative and quantitative data indicated that the intervention was accessible, feasible, and acceptable for participants, Peer Navigators, and service staff alike. Those who received the intervention reported improvements in engaging with services and being better equipped to access services independently, following the intervention period. Participants highlighted the lived experience of their Peer Navigator as helpful in enabling trusting, authentic, and meaningful relationships. Less positively, there was some tension reported between service staff and Peer Navigators which was partly due to a lack of role clarity for service staff, and some crossover between Peer Navigator and existing Support Worker roles. While the feasibility study was not designed to assess the effectiveness of the intervention, participants reported experiencing a range of positive outcomes. For example, crack cocaine use reduced from 52 to 37% over a 6–8-month period, as did gabapentinoid use (34 to 23%). No participants reported an overdose in the last month at follow-up, compared with two participants at baseline. Importantly, mental health outcomes (measured using the Patient Health Questionnaire, PHQ-9, and Generalised Anxiety Disorder, GAD-7) improved overall, and the combined score of these outcomes (PHQ-ADS) demonstrated a reduction in the severity of self-reported depression and anxiety for many. Physical health also improved at follow-up. Finally, retention rates were high for this population, with 78% of participants engaged throughout the intervention period [13].

Given the promising findings from the feasibility study, a randomised controlled trial (RCT) is now required to rigorously explore the effectiveness of the SHARPS intervention on mental health and quality of life, and wider health and social outcomes, and to understand the cost implications of rolling out this intervention across homelessness/social care services.

## Objectives {7}

Our primary aim is to test whether a 12-month peer-led, co-produced, relational, harm reduction, and PIEs-informed intervention (the ‘SHARPS’ intervention) for adults who are experiencing homelessness and problem substance use can improve mental health and quality of life, compared to standard homelessness care. We will do this using a two-arm pragmatic cluster RCT (cRCT). We will also investigate the effects of the intervention on wider outcomes including substance use/harms, risk-taking behaviour, social functioning/support, physical health, service utilisation, and therapeutic alliance. The trial will have an embedded economic and process evaluation to evaluate the cost-effectiveness and transferability of the intervention. As part of the process evaluation, we will examine Peer Navigator outcomes and experiences using a mixed methods approach.

## Trial design {8}

SHARPS is a two-arm pragmatic superiority cRCT involving 20 clusters (cities/towns) in England and Scotland. These 20 clusters will be randomly allocated (1:1) to receive the SHARPS intervention or be assigned to the control group. Within these clusters, a target total of 550 eligible clients will be recruited with support from The Salvation Army (TSA) homelessness services in these areas. Baseline data will be collected from participants prior to receiving the intervention, and follow-up data will be collected 6 and 12 months after the start of the intervention. In control clusters, baseline and follow-up data will also be collected. We will also seek participant consent for future data linkage to longer-term health outcomes.

## Methods: participants, interventions, and outcomes

### Study setting {9}

This is a multi-centre, multi-location, two-arm cRCT set in social care residential and drop-in homelessness services across England (Birmingham, Blackburn, Blackpool, Bradford, Bristol, Coventry, Grimsby, Liverpool, London, Reading, Sheffield, St Helens, Sunderland, Warrington) and Scotland (Aberdeen, Dundee, Edinburgh, Glasgow, Inverness, Perth). Our host organisation for the study is TSA. The research team and TSA have selected services across 20 cities/towns in England and Scotland willing to be randomised to be either control or intervention sites. The city/town will be the unit of randomisation as some have more than one TSA service and, in these instances, the Peer Navigators will work across several services and clients will be recruited across these services.

### Eligibility criteria {10}

Peer Navigators are those delivering the intervention. To be eligible to undertake these roles, all those appointed must have lived experience of homelessness and/or problem substance use. There are a wide range of other competencies laid out in the job descriptions for these roles, including experience of working in the field of alcohol and drugs and/or homelessness and having a qualification in the area of health and social care. In the Peer Navigator role, they explicitly use their lived experience to develop trusting relationships with participants. Having indirect experience such as having a family member with experience of homelessness or problem substance use alone does not make a person eligible to apply for these roles.

The research team will work with TSA service staff to identify potential eligible participants (Table 1). Service Managers and their delegates will create a list of people in the service that meet the inclusion criteria. In advance of the researchers coming to the service, an eligibility meeting will be held between TSA service staff and members of the research team to confirm participant eligibility. When attending the service, researchers will discuss the study with identified eligible people who have indicated interest in taking part. If there are any additional eligible clients that have not been previously discussed, then the researcher will confirm their eligibility with service staff when attending the service. If an individual wishes to participate, they will be asked to sign a consent form (see Additional file 1) and complete the ASSIST Lite screening tool. Once they have signed a consent form, and they meet the threshold of the ASSIST Lite screening tool, they will be recruited into the trial and baseline data collection will be arranged.

**Table 1:** Inclusion and exclusion criteria

*Characteristics of eligible participants*
• Adults aged ≥ 18 years old at the time of consent
• Experiencing homelessness or at risk of homelessness—ETHOS definition [36]
• Self-reported problem substance use (using ASSIST-Lite for health and social care settings increasing [37] risk threshold for any substance except tobacco)
• Not participating in any other homelessness or substance use intervention studies
• Able to speak English
• Able to provide informed consent
• Not pose a safety risk to staff, researchers, or Peer Navigators • Not actively disclosing suicidal intent

### Who will take informed consent? {26a}

Written consent will be obtained by a member of the research team during a visit to the TSA services. Before consent is taken, participants are informed about whether they are in a control or intervention cluster. After obtaining consent, eligibility to take part will be confirmed. Participants will be screened using the ASSIST-Lite tool [37] to confirm that their substance use is of increasing risk. Participants who are screened where substance use is not of increasing risk are excluded. If a participant consents to take part in the study, but their baseline data is not collected within 2 weeks of providing consent, then written consent will be reconfirmed. The participant and researcher will re-initial and re-sign the previously completed consent form to reconfirm consent.

### Additional consent provisions for collection and use of participant data and biological specimens {26b}

Participants are asked to provide optional consent for their data to be used in future data linkage to track longer-term outcomes, and for their data to be included in an anonymised dataset which may be used by other researchers in the future. There are no biological specimens.

## Interventions

### Explanation for the choice of comparators {6b}

Following baseline data collection, participants attending TSA services in clusters assigned to the intervention arm will be assigned a Peer Navigator and participants attending TSA services in clusters assigned to the control arm will receive standard care provided by the service. TSA standard care involves the provision of Support Workers. Support Workers differ from Peer Navigators in that they are not specified as peer workers (with lived experience), do not have a specific focus on tri-morbidity, and are less able to accompany clients to healthcare appointments, benefits or housing meetings, and mutual aid groups, to give a few examples. The process evaluation will systematically describe standard care at baseline, including any variations across all control sites and any changes over the course of the trial.

### Intervention description {11a}

The health technology being assessed is the SHARPS co-produced intervention. The SHARPS intervention is a relational, peer-delivered intervention, informed by harm reduction and PIE principles. The intervention is detailed in the SHARPS intervention guide and training manual, produced for the feasibility study [35]. The intervention guide was co-produced with a range of experts including members of the study team, the feasibility study Peer Navigators, and people with lived experience of homelessness and/or problem substance use. The guide will provide the Peer Navigators with necessary information to carry out their role including practical tools, anticipated challenges, and information about the needs of specific sub-populations.

Ten full-time Peer Navigators will be recruited and employed by TSA on an 18-month contract. All Peer Navigators will have lived experience of homelessness and/or problem substance use and, as a result, are likely to have different experiences of recovery/harm reduction. As part of their role, Peer Navigators will receive training on a range of topics including harm reduction, negotiating professional boundaries as peer workers, therapeutic relationships, PIEs, naloxone administration, and working with those with severe mental health problems. Training will be provided by TSA, the Scottish Drugs Forum, and members of the research team. Additional external training will be utilised where required and available. Considerable support will also be provided to the Peer Navigators in the form of line management in services, support calls every fortnight from the research team in the first 3–6 months of the role (as needed for each individual), and a monthly online group reflective supervision session delivered by a trained peer worker who previously held the role of Peer Navigator in the feasibility trial (WS).

Peer Navigators will work with their clients for 12 months to provide practical and emotional support and facilitate positive changes to their lives (e.g. attending NHS/housing/welfare and other appointments). A fund of £3000 will be available to each Peer Navigator to pay for participant travel, food, hot drinks, clothing, and phone calls, according to participant needs.

### Criteria for discontinuing or modifying allocated interventions {11b}

While participants can choose to discontinue receiving the intervention at any point, their engagement with the SHARPS intervention will not be halted on the basis of either continued problem substance use or abstinence. It may however be necessary to withdraw individual participants if, for example, they behave in violent/aggressive or seriously inappropriate ways towards the Peer Navigator or other member of TSA staff, or if significant capacity to consent to research involvement develops over the life of the study. In these cases, participants will be informed by either the Peer Navigator, Service Manager, or member of the research team that their engagement with the intervention will stop. In all other circumstances, the intervention itself will not be discontinued or modified during the trial period, unless a Peer Navigator was to leave or be unable to continue in post.

### Strategies to improve adherence to interventions {11c}

Peer Navigators will remain in contact with participants even if they leave TSA service. If the participant moves out of the town/city, a discussion will take place between the Peer Navigator, Service Managers, and the research team to determine the appropriateness of the Peer Navigator maintaining the participant on their caseload or not. This will be done on a case-by-case basis. Peer Navigators will maintain contact with participants using text messaging, phone calls, email, or face to face meet ups, depending on participant preferences and availability of technology. In some cases, Peer Navigators may opt to use the budget provided to them to purchase mobile phones or mobile data for their participants (if they do not have one available to them) to increase the likelihood that they will maintain contact throughout the intervention period and support their health and wellbeing journey. Fidelity to the intervention will be assessed using an adapted fidelity tool [38] completed by both the Peer Navigators and their line managers. The Peer Navigators will also keep a training log in order to demonstrate adherence to the required training pathway.

### Relevant concomitant care permitted or prohibited during the trial {11d}

Because the intervention will not replace standard care provided by TSA care settings, participants in the intervention arm may receive some elements of support provided in the standard care settings (e.g. in the form of contact with a Support Worker), in addition to the intervention. As noted above in terms of inclusion/exclusion criteria, those taking part in other homelessness or substance use intervention studies will be excluded.

### Provisions for post-trial care {30}

Towards the end of the intervention, Peer Navigators will work actively with their participants to ensure they are well supported by other members of staff in both TSA services and/or other services in the geographic area post-intervention. Additionally, Peer Navigators will themselves be provided with a range of development opportunities by TSA staff and the research team to help them secure follow-on employment following completion of the trial.

### Outcomes {12}

#### Primary outcomes

The co-primary outcomes are mental health (compositive measure Patient Health Questionnaire Anxiety and Depression, PHQ-ADS) and quality of life (in terms of capabilities) (ICEpop CAPability Measure for Adults, ICECAP-A) at 12 months post-baseline assessment. The co-primary outcomes will be summarised with mean and standard deviation. The analysis will use a mixed effects, repeated measure linear model on the 6- and 12-month outcomes to obtain an adjusted mean difference and 95% confidence interval. The baseline outcome will be included in the model as a fixed effect in addition to the treatment variable, nominal timepoint and country. Random intercepts will be included for participant and cluster.

#### Secondary outcomes

PHQ-ADS and ICECAP-A, EuroQol Quality of Life (EQ-5D-5L) at 6 months post-baseline assessment (12 months is primary outcome measurement above).

At 6 and12 months post-baseline assessment:

- Harmful substance use (Maudsley Addiction Profile (MAP), Leeds Dependence Questionnaire, (LDQ))
- Risk taking behaviours (MAP)
- Social functioning including occupation/education roles (MAP)
- Physical health (MAP, EQ-5D-5L)
- Housing status (self-report housing status)
- Social outcomes, therapeutic alliance with the Peer Navigator (intervention group) and Support Workers (control group), and service accessibility (items from the Social Satisfaction Questionnaire (SSQ))
- Service utilisation (MAP, self-report service utilisation (health, social care, and criminal justice), items from Client Evaluation of Self and Treatment (CEST))
- Relational empathy (Consultation and Relational Empathy measure (CARE))

The quantitative secondary outcomes will be analysed with the same model as the co-primary outcomes and summarised with mean and standard deviation. Categorical outcomes such as service utilisation, housing status, and substance use will be summarised with count and percentage. Any analysis of categorical outcomes will use a logistic or ordered logistic regression to obtain an odds ratio. All estimates of treatment effect will be presented with 95% confidence intervals.

Table 2 below provides an overview of each measure, alongside its use with this trial population (people experiencing both homelessness and problem substance use). It is important to note that, for all of the measures mentioned below, there have been very few studies assessing validity with people experiencing homelessness, although some measures have been validated for use with people experiencing problem substance use. This reflects the wider picture whereby there is very limited evidence on validity for health-related tools more generally for those experiencing homelessness [54].

**Table 2:** Summary of outcome measures

Measure summary	Use with trial population
PHQ-ADS: a validated measure of depression and anxiety comprising the PHQ-9 and GAD-7. There are nine questions about depression and seven about anxiety. It is used extensively in research and clinical practice	PHQ-9 and GAD-7 used successfully in the SHARPS feasibility study [13], and in other studies with this population (for example [39–41]). No evidence regarding validation
ICECAP-A: a validated measure of capability for the general adult population for use in economic evaluations. It comprises five questions about quality of life	There is little evidence that the ICECAP-A has been used or validated with people experiencing homelessness but has been with people experiencing opioid dependence [42]
EQ-5D-5L: a validated measure of self-rated health for use in economic evaluations, which has been used extensively in research. It comprises five questions about quality of life	Has been used in a wide range of studies, including with the trial population (for example [43–45]) but there is limited evidence regarding validity and reliability with this group)
MAP: a structured measure used in treatment outcome research, focusing on drug and alcohol use and related harms. Version edited slightly for relevance to SHARPS by adding questions about overdose and different drugs. It comprises 70 items, covering drug and alcohol use, risk behaviour, housing status, physical and mental health, social functioning, and criminal behaviour	Used successfully in SHARPS feasibility study [13] and in other studies with this population [46–48]. While not validated for use with those experiencing homelessness, the original study showed good reliability with those experiencing problem substance use [49]
LDQ: a validated measure of substance dependence that has been used in research and clinical practice. It comprises 10 questions about drug and alcohol dependence	Widely used in the field of substance use but limited evidence of its use with people experiencing homelessness [50, 51]
SSQ: a validated measure of social satisfaction developed for those experiencing problem substance use. It comprises eight questions about satisfaction with various aspects of life	Has been validated for use among those with problem substance use [52] but no evidence of its use with people experiencing homelessness
CEST: a family of four measures of client needs and progress in substance use treatment: treatment needs, treatment engagement, psychological functioning, and social functioning. Each measure contains 33–36 items	There is evidence of validation with those experiencing problem substance use [53] but not explicitly with people experiencing homelessness
CARE: a validated measure of empathy within a therapeutic relationship and comprises 10 items	Used successfully in SHARPS feasibility study [13], but there are no studies on validity and reliability
Housing status: question about current living situation, adapted from a survey used by a member of the Trial Steering Committee	
Self-report service utilisation (health, social care, and criminal justice): questions about health, social care, and criminal justice resources developed by the SHARPS study leads and study health economists	

#### Economic evaluation outcomes

Collected at 6 and 12 months post-baseline assessment:

- Use of NHS, local government, third sector, and criminal justice services, and associated costs
- Incremental cost per quality-adjusted life year (QALY) and per Year in Full Capability (YFC) gained, with the intervention versus control. QALYs and YFCs will be derived from participant responses to the EQ-5D-5L and ICECAP-A respectively.

#### Health economic evaluation

A full economic evaluation will be carried out using cost-utility analysis. Cost-consequence analysis will also be performed to identify and, where feasible, quantify all costs and outcomes for comparison between the intervention and control group. Costs directly associated with the intervention include salaries of the Peer Navigators and expenses incurred by TSA. Cost of health and social care (primary care, secondary care, community care, medication), criminal justice services, and housing services will also be included in the analysis. Resource use and health outcomes will be measured at baseline, 6and 12 months. Unit costs, required to value reported resource use, will be obtained from published national sources [55–57], study specific estimates, or other published literature. Mixed effects generalised linear models will be used to estimate the mean difference in cost between the SHARPS intervention and control group. QALYs gained, based on the generic EQ-5D-5L health-related quality of life measure [58], and YFC, based on the ICECAP-A capability measure [59], will be used as the measures of benefit. The EQ-5D-5L and ICECAP-A responses will be converted into utility scores using published population tariffs. Incremental QALYs and YFCs for the SHARPS intervention versus control will be estimated using mixed effects generalised linear models, with adjustment for baseline covariates. The health economic analysis will be conducted in accordance with a detailed health economics analysis plan (HEAP) which will be finalised prior to data analysis commencing.

#### Process evaluation

The process evaluation of the SHARPS cRCT will be informed by Normalisation Process Theory (NPT) [60]. There are four components of NPT: coherence (understanding), cognitive participation (buy-in), collective action (making it work), and reflexive monitoring (on-going appraisal) [60]. To ensure transparent data analysis and processes, we will use May et al.’s [61] coding manual. Several theories/frameworks will be drawn on to interpret findings from the process evaluation (e.g. Penchansky and Thomas’ [62] modified access model, and Barker and colleagues’ [63] model of change mechanisms within unidirectional peer support). The process evaluation aims to identify contextual influences on implementation of the Peer Navigator intervention across settings. Specifically, we aim to understand how individuals understood, adopted, or perceived the intervention; participants engaged with/disengaged from the intervention; staff experienced hosting the intervention and being in the control (standard care) settings; the Peer Navigators made sense of their role; and other contextual factors impacting delivery. NVivo software [64] will be used to organise and code data to support the process of qualitative data analysis. The trial Expert by Experience (EbyE) group will participate in data interpretation. We will also take a mixed methods approach to assess intervention fidelity which will include adapting an existing fidelity tool to assess intervention fidelity for a peer-delivered intervention.

#### Equality impact assessment

We will conduct an Equality Impact Assessment (EqIA) to identify how the study may impact on individuals with protected characteristics (age, disability, gender reassignment, marriage and civil partnership, pregnancy and maternity, race, religion or belief, sex, and sexual orientation). The EqIA will inform an action plan where the demographic data collected from participants is monitored to examine equality of access to participating in the study. To fully monitor this, some data will also be collected from Peer Navigators.

### Participant timeline {13}

The participant timeline for enrolment, interventions, and assessments for participants can be found in Table 3.

**Table 3:** Participant timeline for SHARPS study (SPIRIT 2013)

	**Study period**						
	**Enrolment**		**Post-allocation and close-out**				
Timepoint^**^	***-t***_***1***_	**0**	***t***_***0***_	***t***_***3***_	***t***_***6***_	***t***_***9***_	***t***_***12***_
Enrolment:							
Eligibility screen		X					
Informed consent		X					
Cluster allocation	X						
Interventions:							
Intervention: SHARPS				X	X	X	X
Control group (usual care)				X	X	X	X
Assessments:							
Primary outcomes* PHQ-ADS; ICECAP-A*		X					X
Secondary outcomes *PHQ-ADS; ICECAP-A; MAP; LDQ, EQ-5D-5L; SSQ; CEST; hospitalisations*		X			X		X
Secondary outcome; CARE measure					X		X
Economic outcomes* EQ-5D-5L; ICECAP-A; resource use*		X			X		X
*Peer Navigator/Support Worker outcomes ProQOL; JSS*		X					X
*Semi-structured interviews with intervention participants*							X
*Semi-structured interviews with wider staff in both trial arms*							X
*Semi-structured interviews with Peer Navigators*		X					X
*Non-participant observations in both arms*		X			X		X
*Peer navigator diaries (bi-monthly)*		X			X		X
*NoMad measure, Peer Navigators, & intervention staff (online only)*		X			X		X

^**^Note: timepoints are in months (e.g. *t*_*1*_ = 1 month). It should be noted that, as this is a cRCT, clusters taking part in the study are assigned to either the intervention or control arm prior to screening and consenting procedures

### Sample size {14}

The unit of randomisation is city/town (clusters). Each cluster will aim to recruit 25 participants (maximum caseload for a Peer Navigator) in the intervention arm, and 25–35 participants in the control arm. The 20 clusters included in the trial will aim to recruit 550 participants in total, where this is possible: we anticipate an attrition rate of up to 40% in the intervention arm and 50% in the control arm, based on feasibility work/related research. This will result in outcome data on 300 participants (150 in each arm), equating to a mean cluster size of 15. Assuming an ICC of 0.01, this design has 90% power to detect a 0.4SDs effect size at the two-sided 5% level of significance. This equates to a difference of about 0.076 on the ICECAP-A (assuming an SD of 0.19; [42]), and a difference of 5 points on the PHQ-ADS (based on feasibility work SD). Minimally clinically important differences (MCID) reported in the literature for these outcomes are 0.07 for the ICECAP-A [65] and 4 points for PHQ-ADS [66].

### Recruitment {15}

**Fig. 1:**
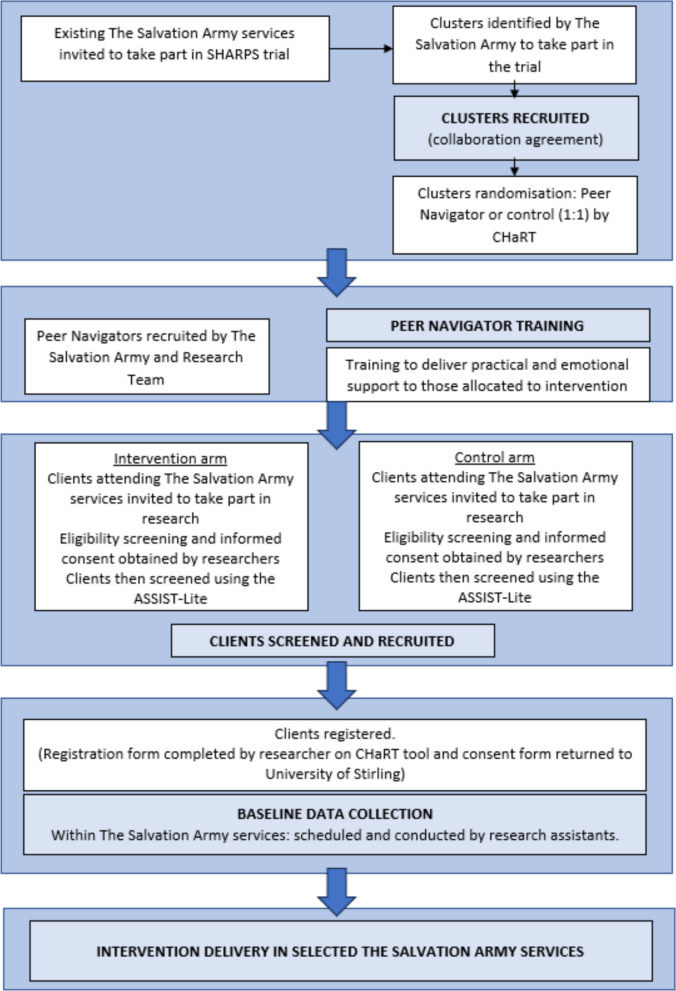
Recruitment pathway, identification of participants, and consent. NB: CHaRT, Centre for Healthcare Randomised Trials

People with problem substance use who are experiencing or at risk of experiencing homelessness will be recruited from TSA services by Service Managers (and/or their delegates) and the research team. See Fig. 1 for details of the full recruitment process.

## Assignment of interventions: allocation

### Sequence generation {16a}

Prior to recruitment of Peer Navigators and participants, clusters (towns or cities) will be randomised to the intervention or control group (1:1) using a computer-generated covariate constrained randomisation algorithm. This approach minimises imbalance on cluster-level covariates which is a potential risk in cRCTs with fewer clusters to optimise balance for clusters based on demographic characteristics, mental health, risk behaviours, homelessness, and number of TSA services. Data will be obtained from area profiles published by the Department for Health and Social Care [67] and the Scottish Public Health Observatory [68]. The most recent data will be used where available. This process will be carried out separately for clusters in Scotland and England.

### Concealment mechanism {16b}

There will be no concealment of allocation at the participant level, as recruitment will take place after clusters have been randomised. Potential participants will be identified by service managers and Peer Navigators in advance of researcher visits, and these staff must be aware of the site’s allocation in order to plan eligibility meetings, distribute trial information, and coordinate researcher visits. As both staff and researchers are required to know the allocation during this process, allocation concealment is not practical in this trial.

### Implementation {16c}

The allocation of clusters to treatment groups will be performed in R statistical software v4.3.1 [69] using code provided by Carter and Hood [70]. The algorithm considers all possible combinations of clusters and obtains a statistic indicating the balance of the demographic characteristics.

The subsequent stages of selecting the allocation and assigning groups to intervention and control will be performed in Stata18 [71]. Choosing the allocation of English clusters will be done by randomly selecting one of the five most optimal allocations. For Scotland, a random selection from the two most optimal allocations will be chosen. Once the English and Scottish allocations are chosen, intervention and control will be randomly assigned to the treatment groups. The initial allocation to treatment groups will be done by the trial statistician. The selection of allocation and subsequent assigning to intervention and control will be done by an independent statistician blinded to treatment groups.

Participants will be enrolled into the study in collaboration between the research team and the TSA services. Each TSA service will identify a list of potentially eligible participants and then a meeting will be held between the research team and the service to go through the eligibility criteria in detail to identify whether each person is eligible. A recruiting researcher will then meet each individual, explain the study to them, and confirm eligibility using the ASSIST-Lite screening tool.

## Assignment of interventions: blinding

### Who will be blinded {17a}

We aim for the researchers employed to collect outcome data at all four time points to be blind to allocation. Researchers will be required to report any occurrences where the allocation of the cluster is revealed to them, or where they believe they have become unblinded to allocation, and this will be recorded by the research team. We will review any occurrences following each data collection point. However, even if we are unable to maintain blinding, i.e. in the event of any researcher unplanned sickness, data collection will go ahead as planned.

Participants, TSA staff, core researchers including statisticians, and Co-Investigators will *not* be blind to intervention allocation. Given the nature of the intervention, it is not possible to blind participants and TSA staff given the presence of Peer Navigators in TSA services will be known to these groups. It is also useful for statisticians to know which participants/clusters are intervention vs control as this can help with safety in terms of monitoring mental health and other outcomes given the vulnerability of the population. To address potential bias concerns, full plans for statistical analysis of outcome data were pre-registered. The research staff leading the statistical analysis (GM & DC) were blind to cluster allocation until the analysis plan was finalised and published on the Centre for Healthcare Randomised Trials (CHaRT) website (https://www.abdn.ac.uk/hsru/what-we-do/trials-unit/statistical-analysis-plans-611.php).

### Procedure for unblinding if needed {17b}

Participants, TSA staff, core researchers including statisticians, and Co-Investigators will *not* be blind to intervention allocation; we aim to blind researchers employed to collect outcome data at all four time points to allocation. There is no requirement for unblinding within this trial.

## Data collection and management

### Plans for assessment and collection of outcomes {18a}

Outcome measures will be assessed at baseline and 6and 12 months post-baseline which will allow us to compare trajectories of outcomes during and after intervention between groups. Excluding baseline data collection, a 12-week window (with 6 weeks either side) will be allowed for each data collection timepoint. Given the challenging life circumstances of the target group, however, we will seek to collect data wherever possible, even if this is outside the 12-week window. The date participants complete data collection will be recorded. If a participant is under the influence at the time of the baseline data collection, or otherwise unable to undertake these measures, this will be rearranged a minimum of three times before they will be withdrawn from the study. The primary measurement point is 12 months post-baseline. In-person electronic data collection via iPads by researchers will be supported via a bespoke database with management tools designed by CHaRT (University of Aberdeen). The researchers carrying out data collection, employed by the University of Stirling, will be trained by the Stirling research team on how to use the data collection tool. They will also be trained on a variety of relevant topics related to working with populations experiencing homelessness and problem substance use. In the unlikely event of technical failure, the data will be collected using paper copies of the questionnaires. Baseline demographic information will also be collected from participants including:

- Participant age, gender, marital status, ethnicity, and disability status
- Education
- Armed forces’ experience and care system experiences
- Housing status

As part of the process evaluation, interviews will be undertaken with staff in a range of roles across both arms (*n* = 40), including commissioning perspectives, and with all the 10 Peer Navigators (at two time points, pre- and post-intervention) to understand experiences of, and views on, the intervention from a range of perspectives, and to collect data on changes in the trial contexts during the study. We will conduct the Normalisation Measure Development (NoMad) questionnaire [72] with a sample of staff (*n* = 4–5) in each intervention setting at three time points (start, middle, and end of intervention, aiming for a total sample size of 100–120) in order to understand the intervention implementation process within services. We will also undertake ‘exit’ interviews with a sample of intervention participants (*n* = 40 at 12 months post-baseline) to understand their experiences of the intervention. Additionally, observations will be undertaken by researchers attending services when collecting outcome measures, to understand the context of TSA services throughout the trial and wider social demographic features of the cluster cities and towns.

### Plans to promote participant retention and complete follow-up {18b}

There are three follow-up data collection points at 6 and 12 months. We will use the 6-month follow-up point, in addition to text messages, emails, and phone calls as opportunities to re-engage participants to reduce overall attrition in this hard to follow-up group (using best practice guidance on retention [73, 74]), balanced with the risk of participant burden and increased trial costs. While not all participants in this trial will have provided contact details, contact details will be requested upon recruitment into the trial. Participants in both the intervention and control groups will be offered a £25 voucher after each quantitative data collection assessment (£25 at baseline and 6 months, and £50 at 12 months, £100 in total), and £25 after the qualitative interview.

### Data management {19}

All data will be collected, stored, and accessed in accordance with the General Data Protection Regulations (GDPR). Only the research team at the University of Stirling who are involved in the day-to-day conduct of the study will have access to participants’ identifiable information stored at the trial office (University of Stirling). Anonymised and pseudonymised quantitative research data will be stored in a secure database hosted by CHaRT at the University of Aberdeen which can only be accessed by a small subset of research study staff. Pseudonymised qualitative data will be stored at the trial office (University of Stirling) and will only be accessed by researchers at the University of Stirling. Paper copies of consent forms will be scanned and saved on the SharePoint site hosted by the University of Stirling, and hard copies securely stored in the trial office (University of Stirling). Data will be entered directly into the secure database by researchers, either at the time of data collection or, if paper copies are used, as soon after the session as possible. The data will be reviewed by the trial office staff to identify data queries and/or missing data. Data queries will be raised to try to ensure a complete and accurate data set. Extensive range and consistency checks will further enhance the quality of the data. Each database user will have their own user account and password. These will not be shared. The trial database has a full audit trail, and every data entry made (or changed) is logged to the specific user.

### Confidentiality {27}

Participant information will remain confidential, unless there are clear reasons to break confidentiality. In addition, before interviews and data collection, participants will be asked to clearly state that they accept and understand limitations to confidentiality. Situations in which breaking confidentiality would occur include disclosures of current or future intent to harm themselves or others. The disclosure from the participant must include clear indication/intent of current (active), potential, or future threats of significant harm towards a specified person or themselves. Significant harm includes, but is not limited to, self-harm, suicidal thoughts or intent, the use of weapons, sexual or physical violence, and general safeguarding concerns for children and vulnerable adults. If such disclosures do occur, this information will be shared with the study leads at the University of Stirling and Service/Programme Managers and/or other relevant senior TSA staff and managers as appropriate who will make decisions and take action as required.

All data will be anonymised or pseudonymised. Each participant will be allocated a unique identifier study code which will be detailed on their consent form. The study team has a data protection/confidentiality agreement with the external transcriber. The audio files will be deleted once they have been transcribed/checked. To protect the identity of study participants, no names will be used in the reporting of the study. We will instead use numerical IDs followed by generic role descriptors such as ‘staff’ and ‘external stakeholder’.

### Plans for collection, laboratory evaluation, and storage of biological specimens for genetic or molecular analysis in this trial/future use {33}

There are no biological specimens.

## Statistical methods

### Statistical methods for primary and secondary outcomes {20a}

Statistical analysis will be conducted according to a detailed Statistical Analysis Plan which has been published on the University of Aberdeen website (please see link to published plan in Sect. 17a). Baseline and outcome data will be described using summary statistics broken down by group. All analyses will be based on the intention-to-treat principle. Primary outcomes will be analysed using a repeated measures mixed effects linear model extended for cRCTs to include a random effect for cluster as well as participant [75]. Models will include a fixed effect for treatment, nominal time, country (Scotland/England), and the baseline outcome score. Treatment effects will be estimated at each time point using a treatment-by-time interaction: the primary measurement time point is 12 months after baseline data collection. A small sample approximation will be applied to the degrees of freedom, given the number of clusters [76]. Secondary outcomes will be analysed in a similar way, with generalised linear models appropriate for the distribution of the outcome. All treatment effects will be presented using 95% confidence intervals. No adjustments for multiple outcomes are planned.

### Interim analyses {21b}

No interim analyses are planned on outcome data collected, and only one final analysis after the final participant has completed follow-up.

### Methods for additional analyses (e.g. subgroup analyses) {20b}

A full economic evaluation will be conducted from a public sector perspective. This will take the form of a cost-utility analysis. We will also conduct a cost-consequence analysis which will identify, and where possible measure, all costs, and consequences (effects) of the intervention, compared to control. Given that the intended effects of the intervention are wider than health effects, we will estimate both an incremental cost per QALY gained and an incremental cost per YFC gained. Capability is measured using the ICECAP-A measure which measures broader well-being.

The process evaluation analysis will draw on NPT [60, 61] to examine contextual influences on implementation across settings: how individuals understood, adopted, or perceived the intervention; how participants engaged with/disengaged from the intervention; how staff experienced hosting the intervention and being in the control (standard care) settings; how the Peer Navigators made sense of role; and other contextual factors impacting delivery. Analysis will be undertaken using the NPT Framework approach and NVivo software will be used to organise and code data to support the process of analysis. All stages of the NPT Framework will be closely followed. To enhance rigour and validity, the trial EbyE group will participate in data analysis/interpretation to act as a form of ‘member checking’ to enhance the validity and trustworthiness of the findings. As part of the process evaluation, we will also take a mixed methods approach to assess intervention fidelity; this will include the use of an adapted existing fidelity index. There are no planned subgroup analyses of the co-primary outcomes.

### Methods in analysis to handle protocol non-adherence and any statistical methods to handle missing data {20c}

The primary analysis will use an unstructured time and covariance structure which gives unbiased treatment effects when outcome data are missing at random (MAR). A MAR mechanism is unlikely to be the case in this population. We will explore the impact of missing data using pattern mixture models under missing not at random assumptions using models for repeated measures data in cluster randomised trials outlined by Fiero et al. [77].

## Plans to give access to the full protocol, participant-level data, and statistical code {31c}

The full protocol is available as a supplement on the funder website (https://fundingawards.nihr.ac.uk/award/NIHR150358). Non-identifiable participant-level data may be available on reasonable request to the Chief Investigator (CI), Professor Parkes (t.s.parkes@stir.ac.uk), if participants agree to this sharing. All statistical analysis code will be freely available on request from CHaRT (DataSharing@abdn.ac.uk), and draft code is available currently in the SAP.

## Oversight and monitoring

### Composition of the coordinating centre, trial steering committee, and EbyE group {5d}

The immediate trial team based in the coordinating centre (Co-CIs (TP, GMac), Deputy CI (HC), Trial Managers (LM, SB, SC), Research Fellow (JB), and Trial Administrator meets every 2 to 3 weeks (Core Team). A Trial Management Group (TMG), comprising the above plus other Co-Investigators, meets approximately every 6 to 8 weeks. A Project Management Group (PMG) comprising of the whole study team meets every 3 to 6 months, depending on the stage of the study. The TMG makes decisions concerning the management of the trial and deals with any challenges as they arise, while the PMG receives updates and makes higher level strategic decisions regarding the trial. The partner organisation TSA is represented at the PMG.

An independent Trial Steering Committee (TSC) has been established to oversee the conduct and progress of the trial. The membership and terms of reference of the TSC will be filed in the trial master file. The TSC is comprised of academics, clinicians, and those with lived experience who are part of the EbyE group. The Chair of the TSC will report to the trial sponsor (University of Stirling) and the trial funder (NIHR). The TSC will meet every 6 months throughout the duration of the trial. The trial Data Monitoring and Ethics Committee (DMEC) also meets every 6 months throughout the trial and is comprised of an independent Chair with subject and methods expertise, an independent statistician, a local commissioner, and a subject expert who was a Peer Navigator in the feasibility study (lived experience expertise). The DMEC will assess the safety and efficacy of the intervention and monitor the overall conduct of the trial.

The SHARPS EbyE group are actively involved in the research process and will actively bring in patient/public involvement to the trial. Responsibilities of the group include reviewing participant materials to ensure they are easy to understand, participating in data interpretation to enhance the validity and trustworthiness of the findings, and collaborating to produce some of the study outputs. The EbyE group will meet every 6 months throughout the trial period and will be chaired by two Co-Investigators (WS & JW). The group will comprise of individuals with lived/living experience of homelessness and/or substance use (and related challenges), including some of those involved in the SHARPS feasibility study as Peer Navigators or EbyE members.

### Composition of the data monitoring committee, its role, and reporting structure {21a}

As above, an independent DMEC has been established to oversee the safety of subjects in the trial. The membership and terms of reference of the DMEC will be filed in the trial master file. The Chair of the DMEC will report to the Chair of the TSC after each meeting is held with their opinion on whether the trial should proceed.

### Adverse event reporting and harms {22}

An adverse events form describing the event (e.g., participant disclosure of harm to self and/or others, harm to Peer Navigator or research staff), and the actions taken, will be completed by the researcher or the Peer Navigator, with only the participant identifier code being recorded on the form. When such disclosures occur, these will be shared with service staff who will conduct a risk assessment, as per their duty of care and TSA responsibilities. If the participant is no longer supported by a TSA service, then a member of the study team will attempt to contact the participant via phone, text, or email within three working days of the disclosure (three attempts will be made). All adverse event forms will be directly entered into the CHaRT secure database by the researcher reporting the adverse event or sent via email to the study lead at the University of Stirling and stored electronically on the secure SharePoint site for the study and then entered into the CHaRT secure database. It is not expected that Serious Adverse Events will result from taking part in this trial due to the nature of the intervention. Any breach of conduct by Peer Navigators or researchers would be taken forward by employing organisations. Any participant deaths will be captured via a Change of Status on the study website. All causes of death will be recorded on the study website where known (this information will be gathered from TSA our trial partner organisation or by applying for death certificates).

### Frequency and plans for auditing trial conduct {23}

The trial office (University of Stirling) and CHaRT colleagues at the University of Aberdeen will monitor aspects of the study on an ongoing basis as described in the study monitoring plan, including review of consent forms, data quality, recruitment, and retention. The trial may also be monitored and audited by the sponsor at any time.

### Plans for communicating important protocol amendments to relevant parties (e.g. trial participants, ethical committees) {25}

Changes to the protocol require the trial office (University of Stirling) to seek permission from the funder, University of Stirling Ethics Committee, and TSA Ethics Committee. The sponsor will be kept updated on changes to protocol. Amendments to the protocol will also be communicated to the full research team. The trial registry will also be updated when required. The TSC, DMEC, and EbyE group will be updated about changes at each meeting held. In addition, advice from TSC and DMEC Chairs will be sought as required in between meetings where items need to be more urgently discussed. The wider study team will be updated at core team, TMG, and PMG meetings and their views will be sought on a range of issues throughout the trial. TSA are also updated at regular trial ‘operational’ meetings.

## Dissemination plans {31a}

The study findings will be disseminated via conference presentations and scientific papers, and shared with relevant organisations including commissioners, the host organisation TSA, and other relevant third-sector organisations, trial participants (where contact details are held), the general public, policy/decision-makers, and various media outlets. We will create a study website and work with the Peer Navigators and EbyE group to create videos and blogs to support dissemination to a variety of audiences. We will also organise a findings ‘roadshow’ to engage a diverse group of stakeholders across the UK.

## Discussion

The trial is a multi-centre, two-arm cRCT. It has been designed to test the effectiveness and cost-effectiveness of a peer-delivered, relational, harm reduction intervention delivered over 12 months for people experiencing homelessness and substance use problems. Previous work has shown the importance of peer support for those experiencing homelessness and problem substance use [14, 18, 20] and, specifically, the SHARPS feasibility study indicated the acceptability and some positive participant outcomes of this intervention including improved service engagement and access to therapy, and reductions in risky drug use [13, 35]. The current study will assess whether the provision of Peer Navigators in TSA settings can improve mental health, quality of life, and related outcomes. The results of the trial will inform future policy and practice in the UK and internationally, providing much needed evidence regarding the effectiveness and cost-effectiveness of peer-delivered interventions for those experiencing homelessness and problem substance use. There may be scope to broaden the context of these roles into other health and social care settings.

A strength of the trial is the continuous involvement of those with lived experience. The intervention was developed by a range of stakeholders, including those with lived experience, and is delivered by Peer Navigators with relevant lived experience. Our EbyE group are integral to the management of the trial and their representation on both the TSC and DMEC will ensure their input is incorporated into all aspects of the trial. Involving a wide range of stakeholders will also ensure timely application of the trial findings.

Overall, SHARPS is an ambitious and original trial but will likely be demanding to deliver operationally and logistically for a number of reasons including the wide geographical spread of clusters, challenges maintaining contact with participants without contact details, and the social care context in which the trial is being conducted which commonly experiences funding cuts to homelessness services.

## Trial status

Protocol V6 was approved and finalised on 10/07/2025. Participant recruitment started on 30/07/2024. Approximate date of anticipated recruitment completion is 12/06/2025.

## Supplementary Information

Additional file 1.

Additional file 2.

## Data Availability

An anonymised dataset consisting of the quantitative data collected during the trial may be available upon request on completion of study and publication of the study results, only if consent to share this data is obtained from all participants. The qualitative data collected during the trial will not be made publicly available given issues in maintaining anonymity of trial participants, Peer Navigators, TSA service staff, and external stakeholders.

## Abbreviations

CARE: Consultation and Relational Empathy Measure
CEST: Client Evaluation and Self Treatment
CHaRT: Centre for Healthcare Randomised Trials
cRCT: Cluster randomised controlled trial
DMEC: Data Management and Ethics Committee
EbyE: Experts by experience
EQ-5D-5L: European Quality of Life 5 Dimensions
ETHOS: European Typology of Homelessness and Housing Exclusion
GAD: Generalised Anxiety Disorder
GDPR: General Data Protection Regulation
ICECAP-A: ICEpop CAPability Measure for Adults
JSS: Job Satisfaction Survey
LDQ: Leeds Dependence Questionnaire
MAP: Maudsley Addiction Profile
MAR: Missing at random
NHS: National Health Service
NICE: National Institute for Health and Care Excellence
NIHR: National Institute for Health and Care Research
NoMAD: Normalisation Measure Development Questionnaire
NPT: Normalisation Process Theory
PIEs: Psychologically Informed Environments
PHQ-ADS: Patient Health Questionnaire Anxiety and Depression
ProQoL: Professional Quality of Life Scale
QALY: Quality-adjusted life year
SD: Standard deviation
SHARPS: Supporting Harm Reduction through Peer Support
SSQ: Social Satisfaction Questionnaire
TSC: Trial Steering Committee
YFC: Years of full capability
