# Supplementary material for: Effectiveness and cost-effectiveness of a peer-delivered, relational, harm reduction intervention to improve mental health, quality of life, and related outcomes, for people experiencing homelessness and substance use problems: protocol for the ‘SHARPS’ cluster randomised controlled trial
Source: Trials. 2025 Dec 13;27:18. doi: 10.1186/s13063-025-09364-x (PMC12790120; doi:10.1186/s13063-025-09364-x)
Supplement: Supplementary file 1 — Additional file 1. [file 13063_2025_9364_MOESM1_ESM.docx]

**Additional File 1. Example consent forms**

**Participant Consent Form (SHARPS intervention participants)**

NICR Approval Number: 2024 16751 Participant number [                          ]

**Research Project Title: Effectiveness and cost-effectiveness of a peer-delivered, relational, harm reduction intervention to improve mental health, quality of life, and related outcomes, for people experiencing homelessness and substance use problems: The ‘SHARPS’ cluster randomised controlled trial.**

| **Please put your initials in each box to confirm that you agree to each statement. For questions that are optional please leave the box blank if you do not wish to give consent for this option.** | |
| --- | --- |
| I confirm that I have read and understood the information sheet (Version 4, January 2025) explaining the above research project and I have had the opportunity to ask questions about the project. |  |
| I consent to complete the ASSIST alcohol and drug screening tool which will determine my eligibility to participate in the above research project. I understand that if my score on the ASSIST screening tool does not meet the threshold of increasing risk then I will not be eligible to participate. |  |
| I consent to complete a range of surveys (measures) about my physical and mental health, substance use, housing status, quality of life, and social support four times during the research project (at the start and after 6, 12 and 15 months). |  |
| I understand that my participation is voluntary and that I am free to withdraw at any time during the data collection session and withdraw my data within 48 hours of it without giving a reason, and without any penalty. I understand that beyond 48 hours it may not be possible to remove my data from the research project. |  |
| I understand that while all information will be kept confidential, the researcher will break confidentiality if they feel that myself or another person is being harmed or at risk of being harmed. |  |
| I consent for my data to be used in future data linkage in order to track longer term outcomes.    (*this is optional – if you do not wish for your data to be used in this way then please let the researcher know).* |  |
| I agree for my data to be included in an anonymised dataset and understand this may be used by other researchers in the future.    (*this is optional – if you do not wish for your data to be included in this dataset then please let the researcher know and we will ensure your data are not included).* |  |
| I consent to work with a Peer Navigator for up to 12 months on issues related to my health, substance use, housing, and other areas as part of this research project. |  |
| **I agree to take part in this research project.** |  |

**Name of Participant**  **Signature:**

**Date:**

**Name of Researcher**  **Signature:**

**Date:**

To enable us to remain in contact with you during the study could you please share your contact details (phone number, email address and address) (this information will be securely stored and only accessible to the core research team).

**Participant Contact Number:**

**Participant Email Address:**

**Participant Address:**

| **If you are consenting to future data linkage, please provide as much of the information below as you can where applicable. Please leave the box blank if you do not wish to give this information.** | |
| --- | --- |
| **First Name** |  |
| **Middle Name(s)** |  |
| **Surname** |  |
| **Previous Surname** |  |
| **Date of Birth** |  |
| **Gender** |  |
| **Sex assigned at Birth** |  |
| **NHS/CHI Number (if known)** |  |

**Participant Consent Form (SHARPS control group participants)**

NICR Approval Number: 2024 16751 Participant number [                          ]

**Research Project Title: Effectiveness and cost-effectiveness of a peer-delivered, relational, harm reduction intervention to improve mental health, quality of life, and related outcomes, for people experiencing homelessness and substance use problems: The ‘SHARPS’ cluster randomised controlled trial.**

| **Please put your initials in each box to confirm that you agree to each statement. For questions that are optional please leave the box blank if you do not wish to give consent for this option.** | |
| --- | --- |
| I confirm that I have read and understood the information sheet (Version 4, January 2025) explaining the above research project and I have had the opportunity to ask questions about the project. |  |
| I consent to complete the ASSIST alcohol and drug screening tool which will determine my eligibility to participate in the above research project. I understand that if my score on the ASSIST screening tool does not meet the threshold of increasing risk then I will not be eligible to participate. |  |
| I consent to complete a range of surveys (measures) about my physical and mental health, substance use, housing status, quality of life, and social support four times during the project (at the start and after 6, 12 and 15 months). |  |
| I understand that my participation is voluntary and that I am free to withdraw at any time during the data collection session and withdraw my data within 48 hours of it without giving a reason, and without any penalty. I understand that beyond 48 hours it may not be possible to remove my data from the project. |  |
| I understand that while all information will be kept confidential, the researcher will break confidentiality if they feel that myself or another person is being harmed or at risk of being harmed. |  |
| I consent for my data to be used in future data linkage in order to track longer term outcomes.    (*this is optional – if you do not wish for your data to be used in this way then please let the researcher know).* |  |
| I agree for my data to be included in an anonymised dataset and understand this may be used by other researchers in the future.    (*this is optional – if you do not wish for your data to be included in this dataset then please let the researcher know and we will ensure your data are not included).* |  |
| **I agree to take part in this research project.** |  |

**Name of Participant**  **Signature:**

**Date:**

**Name of Researcher**  **Signature:**

**Date:**

To enable us to remain in contact with you during the study could you please share your contact details (phone number, email address and address) (this information will be securely stored and only accessible to the core research team).

**Participant Contact Number:**

**Participant Email Address:**

**Participant Address:**

| **If you are consenting to future data linkage, please provide as much of the information below as you can where applicable. Please leave the box blank if you do not wish to give this information.** | |
| --- | --- |
| **First Name** |  |
| **Middle Name(s)** |  |
| **Surname** |  |
| **Previous Surname** |  |
| **Date of Birth** |  |
| **Gender** |  |
| **Sex Assigned at Birth** |  |
| **NHS/CHI Number (if known)** |  |
