# Supplementary material for: Effectiveness and cost-effectiveness of a peer-delivered, relational, harm reduction intervention to improve mental health, quality of life, and related outcomes, for people experiencing homelessness and substance use problems: protocol for the ‘SHARPS’ cluster randomised controlled trial
Source: Trials. 2025 Dec 13;27:18. doi: 10.1186/s13063-025-09364-x (PMC12790120; doi:10.1186/s13063-025-09364-x)
Supplement: Supplementary file 2 — Additional file 2. [file 13063_2025_9364_MOESM2_ESM.pdf]

NIHR HTA

Protocol for Cluster Randomised Controlled Trial of SHARPS

Title: Effectiveness and cost-effectiveness of a peer-delivered, relational, harm reduction intervention to improve mental health, quality of life, and related outcomes, for people experiencing homelessness and substance use problems: The 'SHARPS' cluster randomised controlled trial.

Chief Investigators: Professor Tessa Parkes, SACASR, University of Stirling[1]

Professor Graeme MacLennan, CHaRT, University of Aberdeen[2]

Collaborators/authors: Dr Hannah Carver, University of Stirling[1]

Dr Jennifer Boyd, University of Stirling[1]

Dr Seonaidh Cotton, University of Aberdeen[2]

Dr Suzanne Breeman, University of Aberdeen[2]

Mark Forrest, University of Aberdeen[2]

Dr Rebecca Foster, Edinburgh Napier University[3]

Dr Jake Hawthorn, University of Stirling[1]

Professor Kate Hunt, University of Stirling

Ms Mary Kilonzo, University of Aberdeen[5]

Professor Catriona Matheson, University of Stirling[6]

Professor Margaret Maxwell, University of Stirling[7]

Professor Stewart Mercer, University of Edinburgh[8]

Professor Bernie Pauly, University of Victoria[9]

Mr Wez Steele, Scottish Drugs Forum[10]

Professor Harry Sumnall, Liverpool John Moores University[11]

Professor Graham Scotland, University of Aberdeen[5]

Mr Jason Wallace, Scottish Drugs Forum[10]

Co-ordinating Groups: SACASR, University of Stirling

CHaRT, University of Aberdeen

Protocol Version: V4

This project is funded by the NIHR Health Technology Assessment Programme: **NIHR150358,** Department of Health and Social Care for English Excess Treatment Costs and The Salvation Army for Scottish Excess Treatment Costs.

Trial Registration: ISRCTN11094645

Author affiliations:

[1] Salvation Army Centre for Addictions Services and Research, Faculty of Social Sciences, University of Stirling, Stirling, FK9 4LA.

[2] Centre for Healthcare Randomised Trials, University of Aberdeen, Aberdeen, AB25 2ZD, United Kingdom.

[3] School of Applied Sciences, Edinburgh Napier University, Edinburgh, EH11 4BN.

[4] Institute of Social Marketing and Health, University of Stirling, Stirling, FK9 4LA.

[5] Health Economics Research Unit, University of Aberdeen, Aberdeen, AB25 2ZD.

[6] Centre for Healthcare and Community Research, Faculty of Health Sciences and Sport, University of Stirling, Stirling, FK9 4LA.

[7] Centre for Healthcare and Community Research, Faculty of Health Sciences and Sport, University of Stirling, Stirling, FK9 4LA.

[8] Usher Institute, University of Edinburgh, Edinburgh, EH16 4UX.

[9] Canadian Institute for Substance Use Research, University of Victoria, Victoria, BC, V8P 5C2, Canada.

[10] Scottish Drugs Forum, Glasgow, G1 3LN.

[11] School of Psychology, Liverpool John Moores University, Liverpool, L2 2QP.

Version Control Table for SHARPS Protocol.

Version	Author	Date	Changes
1	Authors listed above	31/05/2024	New document
2	Authors listed above	02/09/2024	• New risk mitigation plan re. recruitment of Peer Navigators added.<br>• New Health Economist on the project added and previous co-investigator removed.<br>• New reconsenting procedure added.<br>• Information on additional means of recording adverse events and change of status directly on the SHARPS website database added.<br>• Skegness removed from list of participating clusters and Grimsby added into the list.
3	Authors listed above	23/10/24	• Information about training for researchers on suicide and self-harm and support added.<br>• Information about number of times data collection will be attempted with a participant added

 © University of Stirling

			• Information about Peer Navigators continuing to work with people who have moved away from the intervention city/town added.<br>• Adverse events procedure updated.<br>• Change to sample size for control clusters.<br>• Eligibility procedure updated.<br>• Information about withdrawal of participants in intervention arms updated.<br>• Safeguarding detail added.<br>• Lone working policy added for Peer Navigators.
4	Authors listed above.		• Adding author to list of authors.<br>• Change to the type of vouchers provided to participants in certain The Salvation Army services where requested.<br>• Addition of a new process where data collectors will request updated contact details from participants at follow-up.<br>• Process for supporting follow-up of participants by The Salvation Army.<br>• Amendment that paper copies of outcome measures will be returned to University of Stirling not University of Aberdeen.<br>• Amendment to the wording in the outcome measures list for adverse events from severity to seriousness.<br>• Removal of the ISEL and specification that we are only using the CEST psychological functioning, treatment engagement and treatment need measure at baseline and follow up data collection.<br>• Update to the adverse events process where the study team will send initials and year of birth of participants in control sites that have disclosed suicidal thoughts to senior management in The Salvation Army so they can take safeguarding processes forward with control site service managers.

			• Addition of a new safeguarding procedure for participants that disclose suicidal thoughts during follow up data collection but are no longer supported by The Salvation Army.<br>• Project Management Group attendees now to include a representative from The Salvation Army, and a representative from The Salvation Army will be removed from Trial Management Group.

Trial key contacts

Chief Investigator
Professor Tessa Parkes
Faculty of Social Sciences
University of Stirling,
Stirling,
FK9 4LA
Email: t.s.parkes@stir.ac.uk

Chief Investigator
Professor Graeme MacLennan
CHaRT, Health Services Research Unit
2nd Floor Health Sciences Building
Foresterhill
Aberdeen
AB25 2ZD
Email: g.maclennan@abdn.ac.uk

Trial Manager/Deputy Chief Investigator
Dr Hannah Carver
Faculty of Social Sciences
University of Stirling
Stirling
FK9 4LA
Email: hannah.carver@stir.ac.uk

Research Fellow
Dr Jennifer Boyd
Faculty of Social Sciences
University of Stirling
Stirling
FK9 4LA
Email: jennifer.boyd1@stir.ac.uk

Senior Trial Manager
Dr Seonaidh Cotton
CHaRT, Health Services Research Unit
3rd Floor Health Sciences Building
Foresterhill
Email: s.c.cotton@abdn.ac.uk

Aberdeen
AB25 2ZD

Deputy Senior Trial Manager
Dr Suzanne Breeman
CHaRT, Health Services Research Unit
3rd Floor Health Sciences Building
Foresterhill
Aberdeen
AB25 2ZD
Email: s.breeman@abdn.ac.uk

Health Economist
Professor Graham Scotland
Health Economics Research Unit
Foresterhill
Aberdeen
AB25 2ZD
Email: g.scotland@abdn.ac.uk

Trial Statistician
Dr David Cooper
Health Services Research Unit
3rd Floor Health Sciences Building
Foresterhill
Aberdeen
AB25 2ZD
Email: d.cooper@abdn.ac.uk

Senior IT Development Manager
Mr Mark Forrest
CHaRT, Health Services Research Unit
University of Aberdeen
2nd Floor, Health Sciences Building
Foresterhill
Aberdeen
AB25 2ZD
Email: mmforrest@abdn.ac.uk

PPI Lead
Mr Jason Wallace
Scottish Drugs Forum
91 Mitchell Street
Glasgow
G1 3LN
Email: jason@sdf.org.uk

PPI Lead
Mr Wez Steele
Scottish Drugs Forum
91 Mitchell Street
Glasgow
G1 3LN
Email: wezs@sdf.org.uk

Table of Contents

1. Trial summary 9

2. **Glossary of terms** 13
3. **Background** 14
 3.1 **Research gap and the SHARPS feasibility study** 15
4. **Aims and objectives** 16
 4.1 **Aim** 16
 4.2 **Research question** 16
 4.3 **Primary objective** 17
 4.4 **Secondary objectives** 17
 4.5 **Process evaluation objectives** 17
5. **Design** 17
6. **Eligibility** 17
 6.1 **Inclusion criteria** 17
 6.2 **Exclusion criteria** 18
7. **Recruitment and randomisation** 18
 7.1 **Recruitment process** 18
 Recruitment of intervention participants 18
 Recruitment of standard care participants 19
 Recruitment of Peer Navigators 19
 Recruitment of intervention participants for qualitative interviews 19
 Intervention setting staff 19
 Standard care staff 20
 7.2 **Participant incentives** 20
 7.3 **Informed consent** 20
 7.4 **Randomisation and blinding** 22
8. **Intervention details** 23
 8.1 **The SHARPS intervention** 23
 8.2 **Control group** 24
9. **Contamination** 24
10. **Withdrawal of consent** 24
11. **Data collection and transfer** 25
 11.1 **Baseline and follow up data** 25
 Primary outcomes 25
 Secondary outcomes 25
 11.2 **Data transfer** 26
12. **Safety** 27
 12.1 **Participants** 27

 © University of Stirling

12.2 Research staff and Peer Navigators 28
13. Statistical, health economic and process evaluation outcomes 29
13.1 Primary outcomes 29
13.2 Secondary outcomes 29
Trial participants 29
Peer Navigators and intervention service staff 29
Peer Navigators & control site Support Workers 29
Safety 30
Economic evaluation 30
The Salvation Army Service observations 30
14. Statistical considerations 30
14.1 Sample size 30
14.2 Frequency of analysis 30
14.3 Primary outcome analysis 31
14.4 Secondary outcome analysis 31
14.5 Interim analyses 31
14.6 Handling missing data 31
14.7 Success and progression criteria 31
15. Health economic evaluation 33
16. Process evaluation 34
17. Experts by Experience group 34
18. Data monitoring 35
18.1 Source data 35
18.2 Data monitoring 35
18.3 Clinical governance issues 35
19. Quality assurance and ethical considerations 35
19.1 Quality assurance 35
19.2 Ethical considerations 35
19.3 Study amendments 36
19.4 Urgent safety measures and serious breaches of good clinical practice 36
19.5 Satellite studies 36
20. Confidentiality 36
21. End of trial and archiving 37
21.1 Definition of end of trial 37
21.2 Archiving 37
22. Statement of indemnity 37

 © University of Stirling

23. Trial organisational structure 38
23.1 Responsibilities 38
Sponsor 38
Project Management Group 38
Trial Management Group 38
Core Trial Group 38
24. Trial oversight 38
24.1 Independent Trial Steering Committee 38
24.2 Data Monitoring and Ethics Committee 38
25. Dissemination 38
26. Publication Policy 39
References 41

 © University of Stirling

1. Trial summary

Title	Effectiveness and cost-effectiveness of a peer-delivered, relational, harm reduction intervention to improve mental health, quality of life, and related outcomes, for people experiencing homelessness and substance use problems: The ‘SHARPS’ cluster randomised controlled trial.	
Short title	SHARPS	
Trial design	A two-arm, pragmatic, cluster randomised controlled trial, with cost-effectiveness evaluation, and embedded mixed methods process evaluation.	
Key inclusion criteria	Individuals attending The Salvation Army Services in one of the 20 included clusters who are over 18 years old; experiencing/at risk of homelessness and self-report problem substance use.	
Planned sample size	550 participants will be recruited from 20 clusters for 90% power to detect 0.076 difference on ICECAP-A and 5 on PHQ-ADS at 12-months (assuming an ICC of 0.01 and 40% attrition in the intervention arm and 50% in the control arm).	
Experimental intervention duration	12 months	
Follow-up schedule	Participants in the trial are followed up at 6 months, 12 months, and 15 months.	
Planned trial period	April 2024 – March 2027	
	Outcomes	Outcome measures
Primary	Mental health<br><br>Quality of Life	PHQ-ADS<br><br>ICECAP-A
Secondary	Harmful substance use<br><br>Risk taking behaviours<br><br>Social functioning<br><br>Physical health<br><br>Social outcomes<br><br>Therapeutic alliance/accessibility and service utilisation<br><br>Relational empathy<br><br>Assess cost-effectiveness of the intervention compared to standard care<br><br>Safety and adverse events	MAP, LDQ<br><br>MAP<br><br>MAP<br><br>MAP, EQ-5D-5L<br><br>SSQ, ISEL<br><br>MAP, CEST<br><br>CARE<br><br>QALYs, Cost, Incremental Cost per QALY, Incremental Cost per Year of Full Capability, Cost and Consequences<br><br>Adverse event form

Economic evaluation	Incremental cost per QALY and Years of Full Capability of the SHARPS 2 intervention versus standard homelessness service care, and include costs to the NHS, local government and criminal justice, costs to the third-sector host organisation, EQ-5D-5L, ICECAP-A, QALYs and Years of Full Capability.<br><br>A cost-consequence analysis which will identify, and where possible measure, all costs, and consequences (effects) of the intervention compared to standard care.
Active intervention	A co-produced intervention delivered by 10 Peer Navigators based in The Salvation Army's third sector homelessness services in 10 town/cities in England and Scotland who provide emotional and practical support to a caseload of up to 25 people for 12 months. Those receiving the intervention may also receive usual care as provided by The Salvation Army services.
Standard care	The Salvation Army services continue delivering usual care, such as support workers signposting clients to other social and health services without being provided with a Peer Navigator.
Economic evaluation	Incremental cost per QALY and Years of Full Capability of the SHARPS 2 intervention versus standard homelessness service care, and include costs to the NHS, local government and criminal justice, costs to the third-sector host organisation, EQ-5D-5L, ICECAP-A, QALYs and Years of Full Capability.<br><br>A cost-consequence analysis which will identify, and where possible measure, all costs, and consequences (effects) of the intervention compared to standard care.
Process evaluation	A mixed methods process evaluation using the MRC guidance on process evaluations of complex interventions and informed by Normalisation Process Theory (NPT). This evaluation aims to 1) investigate transferability to UK public sector settings wider than the third sector and to international settings, 2) examine the 'fit' of the intervention to the context and population, 3) assess recruitment during first 10-months of the trial quantitatively, and 4) assess adherence to/fidelity of the intervention using an adapted existing fidelity index.

Figure 1: Trial summary

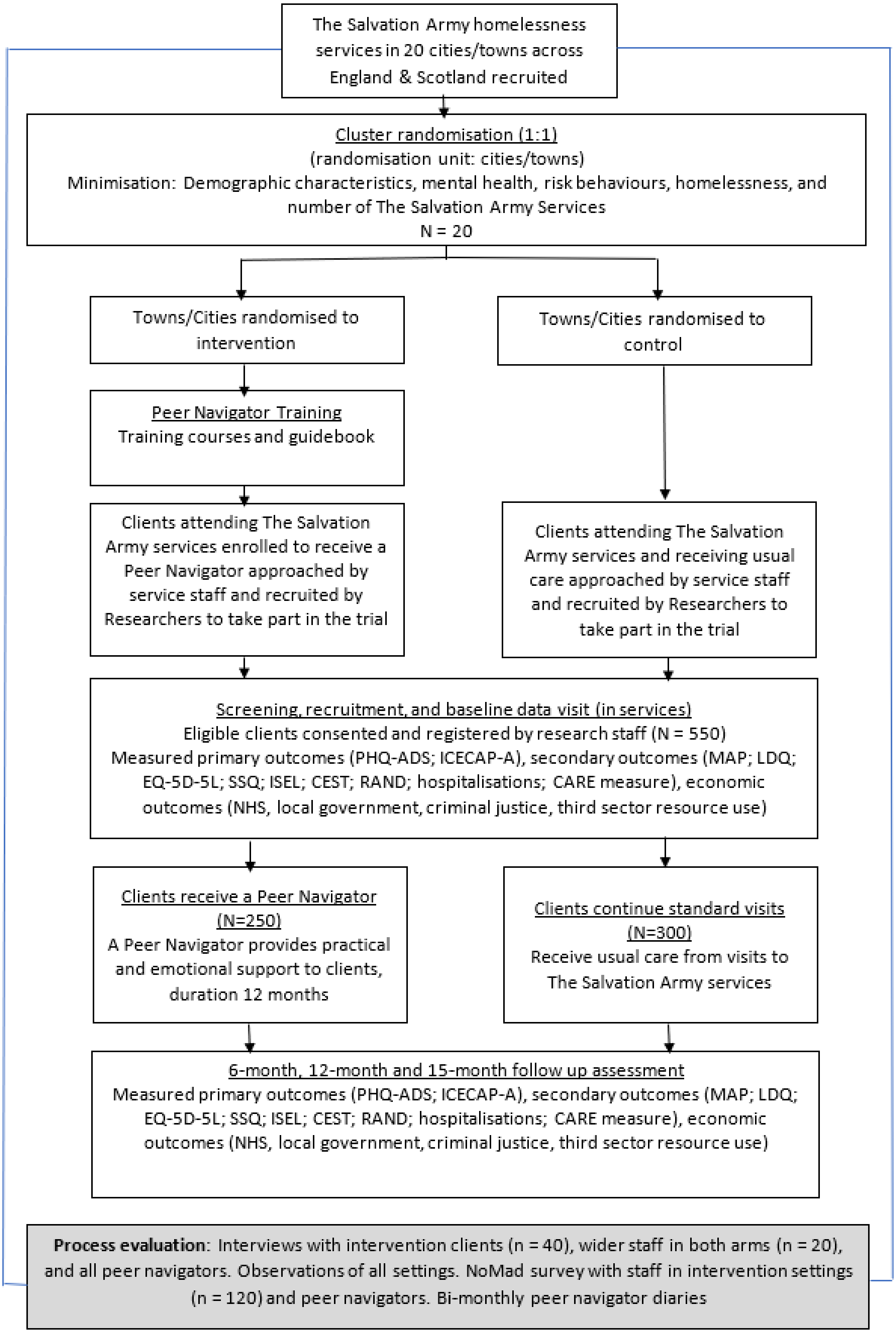

Figure 2: Recruitment pathway: Identification and consent

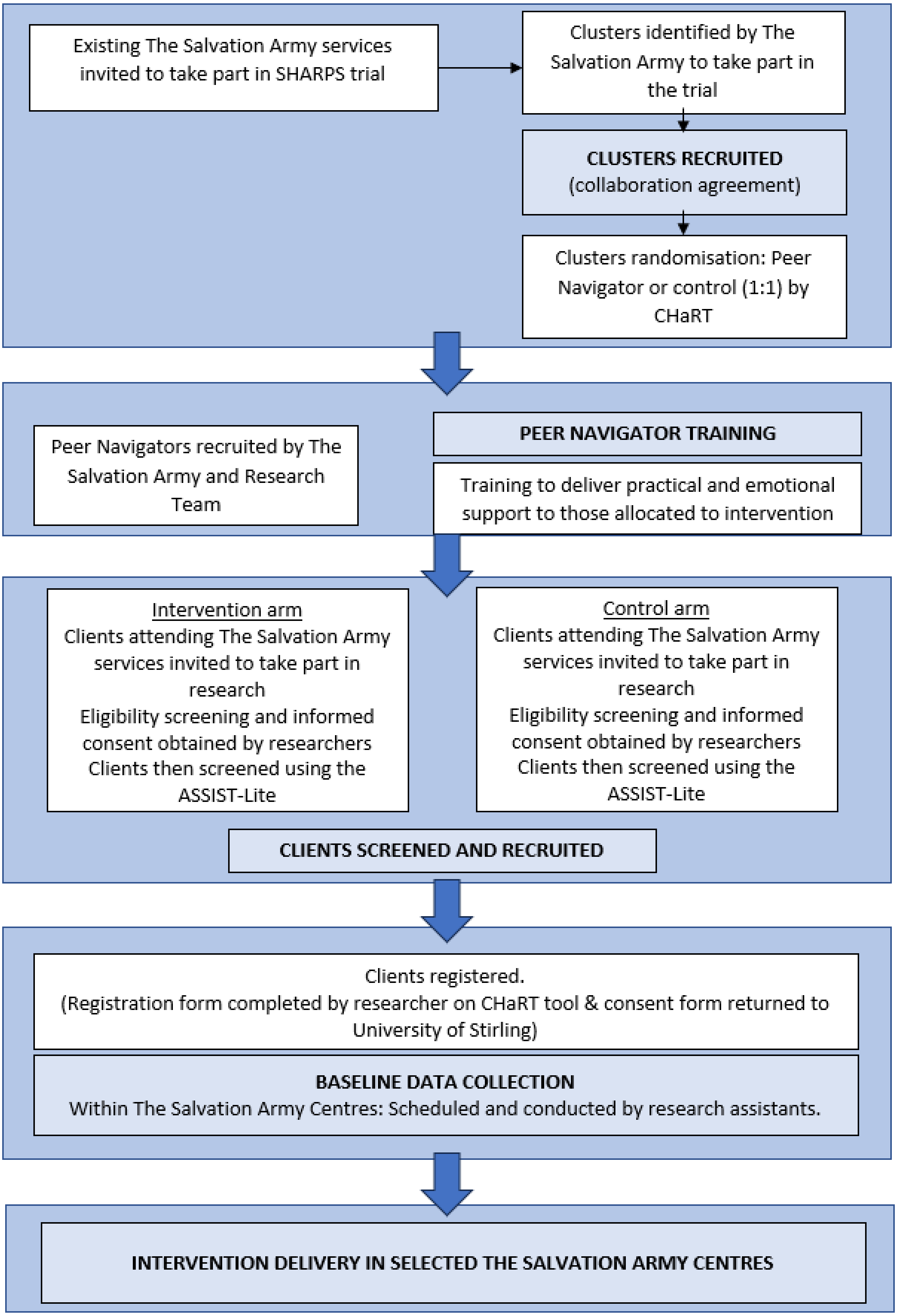

 © University of Stirling

2. Glossary of terms

CARE	Consultation and Relational Empathy Measure
CEST	Client Evaluation and Self Treatment
CHaRT	Centre for Healthcare Randomised Trials
cRCT	Cluster Randomised Controlled Trial
DMEC	Data Management and Ethics Committee
EbyE	Experts by Experience
EQ-5D-5L	European Quality of Life 5 Dimensions
ETHOS	European Typology of Homelessness and Housing Exclusion
GAD	Generalised Anxiety Disorder
GDPR	General Data Protection Regulation
HTA	Health Technology Assessment
ICECAP-A	ICEpop CAPability Measure for Adults
ISE	Interpersonal Support Evaluation
JSS	Job Satisfaction Survey
LDQ	Leeds Dependence Questionnaire
MAP	Maudsley Addiction Profile
MAR	Missing at Random
MRC	Medical Research Council
NHS	National Health Service
NICE	National Institute for Health and Care Excellence
NICR	NHS, Invasive or Clinical Research ethics committee
NIHR	National Institute for Health and Care Research
NoMAD	Normalisation Measure Development Questionnaire
NPT	Normalisation Process Theory
PIEs	Psychologically Informed Environments
PIS	Participant Information Sheet
PHQ-ADS	Patient Health Questionnaire Anxiety and Depression
PMG	Project Management Group
ProQoL	Professional Quality of Life Scale
QALY	Quality Adjusted Life Year
SACASR	Salvation Army Centre for Addiction Services and Research
SD	Standard Deviation
SHARPS	Supporting Harm Reduction through Peer Support
SOPs	Standard Operating Procedures
SSQ	Social Satisfaction Questionnaire
TSC	Trial Steering Committee
YFC	Years of Full Capability

 © University of Stirling

3. Background

People experiencing homelessness are some of the most marginalised and disadvantaged individuals in UK society. Due to unstable housing, they experience significant social and economic challenges (e.g., poverty, social exclusion) which places them at greater risk of a range of acute and chronic health problems, as well as problem substance use and severe mental health challenges [1,2]. The European Typology of Homelessness and Housing Exclusion (ETHOS) definition of homelessness takes into consideration a range of living situations, including rooflessness, houselessness, and insecure or inadequate housing [3]. In 2022/23, in Scotland there were approximately 53,111 people experiencing homelessness [4], while in England there were approximately 271,000 [5]. Tri-morbidity, the co-occurring experiences of poor mental and physical health and problem substance use [6], is common, with use of alcohol and drugs sometimes contributing to someone becoming homeless, and often increasingly as a way of coping with homelessness [7].

For people experiencing homelessness, accessing healthcare and treatment can be particularly challenging due to stigma, negative attitudes from staff, and inflexible services [8,9]. While many people who experience homelessness are registered with primary healthcare, they are not always able to access it when needed, and frequently call on emergency healthcare services to meet their needs [10]. Recent evidence suggests that those who miss primary healthcare appointments are more likely to have mental health problems, including problem drug and/or alcohol use, and are eight times more likely to die prematurely than those who attend appointments [11]. Harm reduction, peer-delivered, and trauma/psychologically informed approaches have shown considerable promise for positively delivering care to these individuals. Therefore, the SHARPS intervention draws together these three conceptual frameworks together to create a co-produced, peer-led, relational, harm reduction, and psychologically informed environments intervention.

Harm reduction is an evidence-based approach to meeting people's needs and reducing the harms associated with substance use without the requirement of cessation of use. It embodies a non-judgemental response to substance use and aims to meet people 'where they are at' [8]. For those who are experiencing homelessness and using substances, harm reduction approaches include: overdose awareness training and naloxone; safe supplies of injecting equipment; drug consumption rooms; and non-abstinence-based housing [8]. Services embodying a harm reduction ethos provide opportunities for developing trusting and reliable relationships between staff and clients and can enable clients to feel accepted develop self-worth and increase access to care [12]. The involvement of peers, those with lived experience of phenomena such as homelessness and/or problem substance use (in this trial this does not include family related experiences), is an important aspect of harm reduction for those who are marginalised, such as those experiencing homelessness and problem substance use [8]. Meaningful involvement of people with lived experience in the design, implementation and evaluation of services is a key principle of harm reduction [13]. Due to space restrictions, we use the terms peer support, peer-led or -delivered services interchangeably, despite key differences.

Several systematic reviews have examined the key outcomes of peer support interventions that are relevant to our trial to assess clinical and cost effectiveness. Evidence shows that peer support can reduce substance use and related harm [9,14] and improve quality of life [9,15,16], mental health [16], social functioning [17], housing/homelessness status [9,16], vocational outcomes [14,16], treatment engagement/acceptability [17,18], access to healthcare [19], engagement with overdose prevention activities [20], retention during COVID-19 isolation [21], and benefit peers themselves [9,22]. One peer support intervention for people with mental health problems showed higher satisfaction with mental healthcare and improved self-reported recovery [23]. A systematic review [24] identified 36 different roles of peers specifically in drugs harm reduction, where involvement of

 © University of Stirling

peers is beneficial in facilitating service engagement and reducing drug-related deaths. In terms of NHS-related policy/practice developments, recent NICE guidelines [25] on integrated services for people experiencing homelessness have highlighted the need to offer peer support, providing role modelling, supporting attendance at appointments, navigating services, forming trusting relationships, and providing advocacy to facilitate continued engagement with services. Challenges for peer workers include power imbalances, high stress, stigma, and exposure to traumatic events [20]. Previous research provides insight into how to best support peer workers, specifically at the intersection of homelessness and problem substance use [26]. Findings indicate that it is critical to provide appropriate training, personal, professional career development opportunities, and nurture relationships between peer workers, and between staff and peer workers [26]. There are also specific challenges that peer workers face when supporting individuals with complex needs including experiencing emotional and stressful situations and setting boundaries [26].

Homelessness settings in the UK are becoming increasingly psychologically/trauma informed, with many services now embedding a psychologically informed environments (PIEs) approach [8,27]. Taking a PIEs approach means understanding how people think, feel, and behave, based on their experiences and environmental factors, including trauma, and using this to inform the design and delivery of appropriate services [8,27]. PIEs involve five key areas: developing greater psychological awareness of people's needs; valuing training and support for all staff, volunteers, and clients; promoting a culture of learning and enquiry, including in service evaluation and improvement; enabling 'spaces of opportunity' which seek to create effective service environments; and a focus on the rules, roles, and responsiveness of the service which focuses on managing and improving relationships [8]. While generally from small or non-randomised studies, there is some evidence that accessing services that are PIEs-informed can lead to improvements in: client mental health and well-being, housing, and behavioural outcomes; engagement with health, substance use, and other care services; as well as reduced involvement with criminal justice and emergency services [8,28–31]. Prior to the trial reported here, there has been a lack of application of PIEs to the field of substance use, a notable research gap. In addition, while peer support appears to be important within a PIEs framework, there is limited evidence regarding the effectiveness of combining the two [8,29,32].

3.1 Research gap and the SHARPS feasibility study

Despite increasing recognition of the value of peer support, and a growing evidence base [14], there are no trials reported in homelessness and substance use. This is in part due to the complexity of conducting trials with this population, given that they are commonly excluded from environments where trials might be conducted [33]. Because trial evidence supports the use of peers within mental health interventions [23,34], there is now a need for an outcomes trial focused on people who experience homelessness and problem substance use to assess effectiveness and cost-effectiveness of peer support interventions.

Our prior feasibility study examined whether a peer-delivered, relational intervention provided by salaried Peer Navigators who received bespoke training was acceptable for people experiencing both challenges, and feasible to deliver in third sector homelessness settings in Scotland and England. The co-produced intervention was created with a team of experts, including those with lived experience, combining evidence on peer support, tri-morbidity, harm reduction, and PIEs [27]. Our study HTA monograph has been published with the findings from the study, alongside three papers [8,35–37]. Our independent Study Steering Committee judged that the SHARPS feasibility study was successful, met its aims, and answered the research questions, with no documented adverse events. They recommended to the NIHR to progress to commission a full next stage trial taking forward the learning to benefit this underserved population with multiple and complex needs.

In the feasibility study, four Peer Navigators were employed to support individuals (n = 68 intervention participants) for up to 12 months based in outreach services and hostels run by three third sector organisations in Scotland and England. Qualitative interviews were conducted with intervention participants, Peer Navigators, and staff in services. Observations were also conducted in both intervention and matched standard care settings. Quantitative outcomes relating to participants' substance use, physical and mental health, and quality of the Peer Navigator relationship, were measured via a 'holistic health check', with six questionnaires completed at two time-points: one at baseline and one at the end of the intervention. The intervention and research processes were found to be acceptable to, and feasible and accessible for, participants, Peer Navigators, and service staff. Participants reported improvements in service engagement and feeling more equipped to access services independently. The lived experience of the Peer Navigators was highlighted as particularly helpful, enabling trusting, authentic, and meaningful relationships to be developed. Some challenges were experienced in relation to the 'fit' of the intervention within some settings, including the crossover between Peer Navigator and support worker roles, lack of role clarity, and tensions between staff and Peer Navigators.

While the SHARPS feasibility study was not designed or powered to produce definitive data on effectiveness, participants did report experiencing a range of positive outcomes, including reductions in drug use and risky injecting practices, and there was an increase in the number of participants receiving opioid substitution therapy. Crack cocaine use was reported as falling from 52% to 37% over a 6–8-month period, as was gabapentinoid use (34% to 23%). While two participants had experienced an overdose in the last month at baseline, no participants reported an overdose in the last month at follow-up. The mean scores for mental health outcomes (Patient Health Questionnaire, PHQ-9, Generalised Anxiety Disorder, GAD-7) improved overall, and the combined score of these (PHQ-ADS) demonstrated a reduction in the severity of self-reported depression and anxiety for many. Physical health also improved at follow-up [8]. Collection of baseline and follow-up measures was successful, with data for n=45 available at baseline and n=30 for baseline and follow up. Retention was also good: 78% engaged throughout the intervention period (and with the research process). Finally, views gathered from qualitative interviews with a sample of participants on randomisation for a future randomised controlled trial (RCT) and data linkage (across health/social care service use) were, overall, positive, with participants seeing the value of conducting a RCT provided that clear information was available on what involvement would entail. When asked whether they would agree to having their data linked, the majority agreed.

Through our feasibility work we have developed a good understanding of the potential benefits of delivering the SHARPS intervention to those experiencing homelessness and problem substance use. A full RCT is now required to fully explore the impacts on outcomes such as mental health and quality of life, and the cost implications of rolling out this intervention across services.

4. Aims and objectives

4.1 Aim

To evaluate the effectiveness and cost-effectiveness (compared with standard homelessness care) of a 12-month Peer Navigator-led, co-produced, relational, harm reduction and PIEs intervention for adults who are experiencing homelessness and problem substance use, for improving mental health, quality of life, and related outcomes, within social care settings.

4.2 Research question

Is a peer-led harm reduction relational 12-month intervention for problem substance use among people experiencing homelessness, delivered in social care settings, effective in improving mental health, quality of life, and related outcomes, and cost-effective, compared to standard care?

 © University of Stirling

4.3 Primary objective

1. Conduct a 2-arm pragmatic cluster RCT (cRCT) across social care homelessness settings in 20 cities/towns in England and Scotland to determine whether the 12-month SHARPS intervention improves mental health and quality of life (co-primary outcomes), compared to standard homelessness care, in adults who are experiencing homelessness and problem substance use.

4.4 Secondary objectives

1. Compare secondary outcomes, including substance use/harms, risk-taking behaviour, social functioning/support, physical health, homelessness, therapeutic alliance, and relational empathy;
2. Undertake a cost-utility and cost-consequence analysis of the SHARPS intervention.

4.5 Process evaluation objectives

1. Conduct a process evaluation guided by MRC guidance and Normalisation Process Theory (NPT) to examine transferability, context, and intervention 'fit';
2. Assess intervention adherence/fidelity via mixed methods;
3. Examine Peer Navigator outcomes/experiences via mixed methods.

5. Design

The study is a 2-arm pragmatic cRCT delivered across 20 clusters (cities/towns) in England (Birmingham, Blackburn, Blackpool, Bradford, Bristol, Coventry, Grimsby, Liverpool, London, Reading, Sheffield, St Helens, Sunderland, Warrington) and Scotland (Aberdeen, Dundee, Edinburgh, Glasgow, Inverness, Perth), with embedded economic and process evaluations involving mixed methods including qualitative interviews (participants, service staff, Peer Navigators), and participant consent for future data linkage to longer term health outcomes. Twenty-five participants from each intervention cluster and between 25-35 participants from each control cluster will be recruited to the study (to address the potential for greater attrition in these sites).

Outcomes will be collected at 6-, 12- and 15-months post baseline data collection. Outcomes from the Peer Navigators will be collected at 12- and 15-months post baseline data collection. A mixed methods approach will be used throughout the trial.

6. Eligibility

6.1 Inclusion criteria

Cluster inclusion criteria: The Salvation Army homelessness services in a town or city collectively willing to be randomised to control or intervention. All clusters were selected by The Salvation Army and the study team prior to the trial start date.

Participant Inclusion criteria: >18 years and experiencing/at risk of homelessness (ETHOS definition [3]); self-reported problem substance use and meet the pre-determined cut-off based on the ASSIST-Lite screening tool; able to provide informed consent. Prospective participants will complete the ASSIST-Lite screening tool for health and social care services, if they meet the cut-off (scored as increasing risk for any substance including alcohol) then they will be eligible to take part in the study. The level and nature of problem substance use will vary between individuals. It is anticipated that, as with our feasibility work, most participants will be experiencing problem substance use that is severe and has an ongoing and substantial impact on their daily lives. Potential participants who are under the influence of alcohol/drugs affecting their immediate ability to consent (but who do not

© University of Stirling

have long-term/permanent cognitive impairment) will be re-approached to participate and consent one or two days later. Those who are under the influence will be mentioned to service management from the point of view of safeguarding to ensure that they are provided with the appropriate support. If a participant is under the influence at the time of the data collection, or otherwise unable to undertake these measures, this will be rearranged a minimum of three times before they will be withdrawn from the study.

6.2 Exclusion criteria

Participant exclusion criteria: Currently receiving any other homelessness and/or substance use interventions (outside of The Salvation Army usual care) or participating in the active intervention phase of intervention studies; inability to give clear informed consent due to serious mental illness or cognitive impairment; actively suicidal; posing a safety risk to staff members/Peer Navigators; non-English speaking (due to the intervention involving Peer Navigator relationship and they will not necessarily have languages other than English).

Participation requires ability to provide consent which might be compromised for those with very serious mental illness or cognitive impairment. For people who experience homelessness, severe mental illness is not common (6% bipolar disorder, 6% schizophrenia; [38]) however, prevalence of cognitive impairments can be higher than the general population [39], therefore, clinical expertise from collaborator Dr Jake Hawthorn will support the creation of a recruitment consent protocol.

7. Recruitment and randomisation

7.1 Recruitment process

Recruitment of intervention participants

The research team will work with the Peer Navigators and The Salvation Army Service Managers (and their delegates, which may include Programme/Project Managers and/or Specialist Support Workers/experienced Support Workers) to identify potential participants. Service Managers, Peer Navigators and their delegates will create a list of people in the service who meet the inclusion criteria. In advance of the researchers coming to the service, an eligibility meeting (via MS Teams) will be held between Service/Programme/Project Managers/Peer Navigators and the Cluster Leads/Chief Investigator/recruiting Researcher. These meetings are designed to build productive relationships and will go through the eligibility criteria in detail, identify how many potential eligible participants there are likely to be, discuss the process of sharing the Participant Information Sheets (PIS) with participants in advance of researchers arriving in service, address any questions the service staff have, and set up the arrangements for the visits. Following the eligibility meeting, if individuals meet the study inclusion criteria a staff member in the service will tell them about the study and provide them with a copy of the PIS prior to the researchers being in the service. If it is not possible to hold an eligibility meeting prior to researchers visiting a service then researchers will work with service staff in person to identify eligible potential participants. When the researchers are in the service, if an individual is interested, a researcher will discuss the study with them, going through the PIS and answering any questions they may have. If they wish to participate, they will be asked to sign a consent form and then their substance use will be checked using the ASSIST-Lite screening tool. Once they have signed a consent form, and they meet the threshold of the ASSIST-Lite screening tool, they will be recruited into the trial and baseline data collection will be arranged. Researchers will visit The Salvation Army services and aim to recruit 25 participants per cluster, under the guidance of the research team. There may be times when there are additional potential participants who were not discussed at the initial eligibility meeting, but who have been identified during the time in which the researchers are in the service. At this point, the recruiting researcher will discuss the potential participant with the Service Manager or their delegate and check they meet

 © University of Stirling

the criteria and then confirm this with the Cluster/Study Leads. Once the participant has been recruited into the study and completed the baseline data collection they will be added to the Peer Navigator's caseload.

Recruitment of standard care participants

The research team will work with The Salvation Army Service Managers (and their delegates, which may include Programme/Project Managers and/or Specialist Support Workers/experienced Support Workers) to identify potential participants. Service Managers and their delegates will create a list of people in the service who meet the inclusion criteria. In advance of the researchers coming to the service, an eligibility meeting (via MS Teams) will be held between Service/Programme/Project Managers and the Cluster Leads/Chief Investigator/recruiting researcher. These meetings are designed to build productive relationships and will go through the eligibility criteria in detail, identify how many potential eligible participants there are likely to be, discuss the process of sharing the PIS with participants in advance of researchers arriving in service, address any questions the service staff have, and set up the arrangements for the visits. Following the eligibility meeting, if individuals meet the study inclusion criteria a staff member in the service will tell them about the study and provide them with a copy of the PIS prior to the researchers being in the service. If it is not possible to hold an eligibility meeting prior to researchers visiting a service then researchers will work with service staff in person to identify eligible potential participants. When the reearchers are in the service, if an individual is interested, a researcher will discuss the study with them, going through the PIS and answering any questions they may have. If they wish to participate, they will be asked to sign a consent form and then their substance use will be checked using the ASSIST-Lite screening tool. Once they have signed a consent form, and they meet the threshold of the ASSIST-Lite screening tool, they will be recruited into the trial and baseline data collection will be arranged. Researchers will visit The Salvation Army services and aim to recruit at least 30 participants per cluster (up to 35 in some clusters to offset control clusters with fewer than 30 participants), under the guidance of the research team. There may be times when there are additional potential participants who were not discussed at the initial eligibility meeting but who have been identified during the time in which the researchers are in the service. At this point, the recruiting researcher will discuss the potential participant with the Service Manager or their delegate and check they meet the criteria and then confirm this with the Cluster/Study Leads.

Recruitment of Peer Navigators

The Peer Navigators will be employed by The Salvation Army as part of the study and the research team will already have their contact details because of this. They will be contacted to arrange data collection via interviews, diaries, and outcome measure data collection. There will be one Peer Navigator recruited for each cluster in the intervention arm.

Recruitment of intervention participants for qualitative interviews

The research team will work with Peer Navigators to identify potential participants for interviews. The Peer Navigators will create a list of people who would be willing to participate in an interview and ask their permission for their details to be shared with the research team. If someone is interested and agrees for their details to be passed on, a Research Assistant will be in touch to provide more information about the interview.

Intervention setting staff

Participants will be those working in the intervention settings (Service Managers, Support Workers etc). Service Managers will be asked to provide a list of relevant staff who will be eligible to participate and provide their email addresses, including themselves potentially. Service managers will inform staff that they intend to share email addresses with the study team at which point they will

be able to object to having their email address shared. The research team will then contact relevant individuals to ask if they would like to participate in an interview, and for them to complete the NoMAD survey [40].

Standard care staff

Support Workers working in the standard care settings will be recruited to take part in outcome measure data collection (the same measures as being conducted with the Peer Navigators). Service Managers will be asked to provide a list of relevant staff who will be eligible to participate and provide their email addresses, including themselves potentially. Service managers will inform staff that they intend to share email addresses with the study team at which point they will be able to object to having their email address shared. The research team will then contact relevant individuals to ask if they would like to complete the outcome measures.

7.2 Participant incentives

Participants in both the intervention and control groups will be offered a £25 Love2Shop voucher after each quantitative data collection assessment (£100 in total if all measure points are successfully achieved) as a 'thank you' for participation as per NIHR rates [41]. Intervention participants who take part in an interview for the process evaluation will be offered a £25 Love2Shop voucher. If requested by The Salvation Army service manager we will offer a £25 clothing only voucher (e.g., Primark, Matalan) as an alternative to a Love2Shop voucher. We comply with INVOLVE guidance and do not consider that a £25 voucher would attract a person to take part in this study if they do not wish to, despite the circumstances of considerable hardship.

7.3 Informed consent

We will ensure that participants are recruited in a fair and ethical manner, in line with equality, diversity, and inclusion practices and good research practice. Participants will be asked to provide written informed consent prior to eligibility screening using the ASSIST-Lite tool and baseline data collection, and consent will be checked and recorded at each subsequent data collection timepoint. Participants who are not able to read or write (but who have capacity) can agree to take part in the study. In such cases, the study team will provide them with written literature about the study and read and discuss this information with the potential participant. There will also be a discussion about the support networks that the patient has available to facilitate their participation in the study. If the potential participant is fully informed and wishes to take part in the study, they will be asked to sign or make their mark on the consent form. Their agreement to take part in the study should be witnessed by the Service Manager who will be independent from the research team. While most participants in the SHARPS feasibility study did not report any literacy difficulties, we will ensure that all materials are explained verbally to all participants to ensure they are fully aware of what the study/interviews entail.

All participant materials will be checked by trial Experts by Experience (EbyE) group to ensure they are easy to understand. These materials have been based on those used in the SHARPS feasibility study which were thoroughly reviewed by our EbyE group and learning from that study. All final copies of study materials will be provided to the University of Stirling's NHS, Invasive or Clinical Research (NICR) ethics committee and logged in the trial files. Intervention and control group participants will receive a PIS detailing the study and what is required from them, a consent form, and debrief sheet which will provide participants with information about the study once the study is complete. Both groups will be asked to consent to data collection within the study. In addition, intervention participants will consent to the intervention itself specifically within the general consent form with information on the intervention clearly spelled out in the PIS.

Exit qualitative interviews will be conducted with a sub-sample of n=40 (n=20 at 12 months and n=20 at 15 months) intervention participants. Those participating in an exit interview will receive a separate PIS, consent form and debrief sheet. This is only relevant for the intervention participants.

Quantitative data collection will occur at baseline, and 6, 12, and 15 months after recruitment for both intervention and control groups.

Participants will be able to leave the trial at any time but will not have their trial status changed by researchers (or by the Peer Navigators) based on either continued substance use, abstinence, or lack of engagement with their Peer Navigator (loss of contact is different from lack of engagement see below). Withdrawal processes will be clearly explained in the PIS and participants will be provided with information regarding how to contact the study team, if required. Participants may become ineligible, however, and need to be withdrawn from the trial. This may relate to changes in behaviour (for example displaying violence/aggression towards the Peer Navigator that was not apparent at recruiting into the trial), loss of contact over a period of time, or in relation to capacity to consent to trial conditions. In these cases, participants will be informed by the Peer Navigator, Service Manager/Programme or Project Manager, or member of the research team, that they are no longer able to continue in the trial and a Change of Status form will be completed. Participants in the intervention arm may also move away from the city/town during the course of the study. In such cases they may still be able to continue in the trial and work with the Peer Navigator. The most appropriate option will be discussed between the Peer Navigator and participant and the Peer Navigator, Service/Programme/Project Manager and Study Leads.

In addition, as per NIHR guidance, we will ask study participants to consent to long-term follow-up (e.g., beyond the outcomes to be collected in the proposed study) using routinely collected data, and appropriate linkage to allow these data to be best used. This activity (long-term follow-up) will not be conducted during this three-year trial but instead it is purely ascertaining consent for such linkage to be conducted at a future point. Participants will be asked about whether or not they consent to data linkage and will be informed that this is optional – they can say no and still be involved in the trial. For those who say yes, basic details to facilitate data linkage in the future will be collected.

As per developing practice around open science, we will also ask intervention and control group participants if they consent to sharing their anonymised data with other researchers in the form of the final quantitative dataset at the end of the study. We must balance the need to ensure recruitment targets are met with this desire to comply with best practice in the field. If client participants are not comfortable with sharing their data, we will revise our plan to do this because it is more important to be sensitive to the needs of this client group. The client group is likely to have had experiences that may make them suspicious of data sharing, despite the commitment to fully anonymising the dataset. We will provide a box on the consent form which indicates that sharing their details via open science will be optional and then the research team will decide about proceeding with the possibility of the open science dataset being shared once all participants are recruited. We will not be sharing the qualitative datasets due to potential small numbers and challenges anonymising the data.

In cases where someone has previously consented to take part in the study, but they did not complete the baseline outcome measures, the researcher recruiting participants at the next visit to the service will have to reconfirm in writing that the participant consents to take part in the study (if the first time of consent was longer than two weeks ago). The researcher will update the participant’s original consent form by having the participant re-sign and re-date the form, the researcher will also have to re-sign, date, and initial the form. A note will then be added to the participant’s record on the SHARPS study data collection tool to reflect that they have had their consent reconfirmed in writing and the date of written reconsent.

7.4 Randomisation and blinding

We will allocate clusters to intervention and control group using covariate constrained randomisation. This approach minimises imbalance on cluster level covariates, which is a potential risk in cRCTs with fewer clusters. We will use Carter and Hood's [42] algorithm to optimise balance on the following for clusters located in Scotland:

- *Demographic characteristics*: population size, % population identify as white, % population income deprived, % working age population employment deprived.
- *Mental health:* suicide rate per 100,000.
- *Risk behaviours*: adults in drug treatment per 1000, adults in alcohol treatment per 1000, adults in co-dependency treatment per 1000, drug-related deaths per 100,000, alcohol-specific deaths per 100,000, alcohol-related hospitalisations per 100,000.
- *Homelessness*: households assess as homeless per 1000 households, households in temporary accommodation per 1000 households.
- Number of The Salvation Army services.

And for clusters located in England we will use the following:

- *Demographic characteristics*: population size, % population identify as white, deprivation score (IMD 2019).
- *Mental health*: suicide rate per 100,000.
- *Risk behaviours*: adults in drug treatment per 1000, adults in alcohol treatment per 1000, drug-related deaths per 100,000, rate of alcohol dependency per 100, proportion of opioid and/or crack-cocaine users not in treatment.
- *Homelessness*: relief duty owed per 1000.
- Number of The Salvation Army services.

These data will be obtained from area profiles published by the Office for Health Improvement and Disparities/Public Health Scotland. The most recent data will be used where available.

We aim for the researchers employed solely to collect outcome data at all four time points to be blind to allocation. Full details of the data collection process can be found in section 11 of the protocol. In an attempt to blind Researchers collecting outcome data, all participants will be recruited and consented into the study by members of the research team who are not involved in the quantitative data collection. We will also provide guidance to The Salvation Army staff to ensure they are aware that they must not discuss the study with Researchers. Researchers will be required to record and report any occurrences where the condition of the cluster is revealed to them. We will review any occurrences following each data collection point. However, even if we are unable to maintain blinding of Researchers, data collection will go ahead as planned.

Intervention and control/usual care participants, Peer Navigators, The Salvation Army staff, and core research staff including statisticians and health economists will not be blinded to intervention allocation. Given the nature of the intervention, it is not possible to blind participants and The Salvation Army staff as the presence of Peer Navigators in these services will be known to these groups. It is also useful for statisticians to know which participants are intervention vs. control as this can help with safety in terms of monitoring mental health and other outcomes given the vulnerability of the population. To address potential bias concerns, full plans for statistical analysis of outcome data will be pre-registered (the practice of registering hypotheses, methods, and planned analyses online prior to any data analyses).

8. Intervention details

8.1 The SHARPS intervention

The health technology being assessed involves the provision of the SHARPS co-produced intervention. This is relational, peer-delivered intervention, informed by harm reduction and PIEs principles. The intervention itself is laid out in the SHARPS intervention guide and training manual that were produced as part of our feasibility study. The intervention guide was co-produced at a full day meeting with a range of experts including members of the study team, the (original feasibility study) Peer Navigators, experts working in the field, and other individuals with lived experience of homelessness and/or problem substance use. The guide provides the Peer Navigators with most of the necessary information to carry out their role, including practical tools, anticipated challenges, and information about the needs of specific sub-populations.

Ten full-time Peer Navigators will be recruited and employed by The Salvation Army on 18-month contracts for 40 hours per week. All Peer Navigators will have lived experience (not just family experience) of problem substance use and/or homelessness and are likely to have different experiences of recovery/harm reduction. We will be using our experience/learning from the feasibility study and best practice internationally to get the right balance in relation to the nature of lived experience/time since active problem substance use (if any). It should be noted that no Peer Navigator experienced a substance use relapse during the SHARPS feasibility study but all of them experienced stress related to the nature of the work. They received considerable support via line management in services and from the reflective supervision that was put in place. This will be maintained in the SHARPS cRCT.

We will be using a carefully constructed recruitment process to ensure that we acknowledge both strengths and potential vulnerabilities when recruiting to these roles. We will be involving experts in this field (our co-investigators from Scottish Drugs Forum), who have run a national addiction worker training programme for around 20 years, to help us navigate this important area of Peer Navigator recruitment. In addition, one of the co-investigators on the study was employed as a Peer Navigator during the SHARPS feasibility study and will be involved in the recruitment of the Peer Navigators (Steele, now employed by Scottish Drugs Forum). He will bring his own learning from being recruited and employed as a Peer Navigator to this process.

The Peer Navigators will work intensively with clients for a 12-month period to facilitate changes to their lives, including attending NHS/housing/welfare etc appointments. Each Peer Navigator will provide practical and emotional support to clients on their ‘caseload’. As per the SHARPS feasibility study, a fund (£3,000) will be available to each Peer Navigator to pay for participant travel, food, hot drinks, clothing, and phone calls, according to participant needs. Engagement with participants will be ongoing and have a clear beginning, middle, and end. This is very important for endings: towards the end of the intervention the Peer Navigators will work actively with clients to ensure they are well supported by other members of staff in intervention settings/other services post-intervention.

As part of their role, Peer Navigators will receive training on a range of topics including harm reduction, trauma and psychologically informed care, motivational interviewing, negotiating professional boundaries, therapeutic relationships, and naloxone administration. Training will mainly be provided by Scottish Drugs Forum and via The Salvation Army as part of the Peer Navigators’ induction to the organisation. Workplace supervision will be provided by The Salvation Army Service Managers who will line manage the Peer Navigators. This will be supplemented by monthly online group reflective supervision sessions delivered by a trained peer worker (Steele, Scottish Drugs Forum).

 © University of Stirling

We plan one Peer Navigator per cluster intervention city/site with the possibility for some limited paired working across Peer Navigator (intervention only) clusters, where feasible. We will endeavour to link the Peer Navigators to their counterparts in other trial intervention sites services geographically closer to them for additional peer-to-peer support between Peer Navigators in addition to the reflective sessions noted above.

8.2 Control group

The Salvation Army services within clusters assigned to the control arm will not have Peer Navigators. Instead, participants will receive the local care (standard care) pathway within The Salvation Army services. While standard care within these settings may vary between areas it usually involves having support workers help those attending services with a range of issues for example with housing applications, with contacting relatives, or wider support services. The qualitative process evaluation will systematically describe standard care at baseline, including any variations across all control sites, and any changes over the course of the trial.

9. Contamination

We have made efforts to reduce the risk of contamination between intervention and control sites in this trial. Given that the Peer Navigators will work across services within their area we have opted to randomise cities and towns rather than the services themselves. We will also monitor whether participants move location during the trial period.

10. Withdrawal of consent

Participants will be able to withdraw from the intervention at any time. Where participants wish to withdraw, clarification will be sought as to whether they wish to withdraw from receiving the intervention, short-term data collection (6 months), medium-term data collection (12 and 15 months) or a combination. All changes in status, except for complete withdrawal of consent, means the participant is still followed up for all trial outcomes wherever possible. Changes in status will be recorded on an electronic form and returned to the trial office in Stirling by email. All data collected up to the point of complete withdrawal are retained and used in the analysis. In addition, the Study Leads may discontinue a participant from the study at any time if they consider it necessary for any reason, for example if they later become ineligible due to changes in behaviour, loss of contact, or capacity. When participants initiate withdrawal from the study, they will be asked to provide a reason which will be recorded on the change of status form.

Following informed consent, if a participant loses capacity, the consent given when capable remains legally valid. In such circumstances, a decision needs to be made, in conjunction with the participant and any family or carers/Service Managers, in relation to ongoing participation in the study.

Participants will not be withdrawn by research staff based on either continued substance use or abstinence. Disengagement or limited interactions with the assigned Peer Navigator will also not be cause for withdrawal from the trial (as long as some contact is maintained to allow data collection for the trial). Efforts will be made to collect data unless participants express a wish to withdraw completely from the trial.

Clusters will not have the option to withdraw as they are designated sites as selected by The Salvation Army to take part in the trial. The only exception to this would be if all The Salvation Army services within the cluster are decommissioned, in which case we would put in place our advance contingency measures depending on how far into the trial period this takes place. The study team will be actively tracking the risk of potential decommissioning before and through the course of the study.

11. Data collection and transfer

A summary of data collection measures and timepoints are provided in Table 1.

Table 1: Timing of measurements/process evaluation data collection

	Baseline	6m	12 m	15 m
Primary outcomes (PHQ-ADS; ICECAP-A)	✓	✓	✓[a]	✓
Secondary outcomes (MAP; LDQ; EQ-5D-5L; housing status; therapeutic alliance; SSQ; CEST psychological functioning; CEST treatment engagement; CEST treatment need; service utilisation)	✓	✓	✓	✓
Secondary outcome (CARE Measure)		✓	✓	✓
Peer Navigator/Support Worker outcomes (ProQOL, JSS)	✓		✓	✓
Semi-structured interviews with intervention participants			✓	✓
Semi-structured interviews with staff in both trial arms			✓	
Semi-structured interviews with external stakeholders in intervention arm			✓	
Semi-structured interviews with Peer Navigators	✓			✓
Non-participant observations in both arms	✓	✓	✓	
Peer Navigator diaries (bi-monthly)	✓	✓	✓	✓
NoMad measure, Peer Navigators, intervention staff (online only)	✓	✓	✓	

[a] Primary outcome is assessed at 12 months; PHQ-ADS and ICECAP-A at 6 and 15 months are secondary outcomes.

11.1 Baseline and follow up data

<u>Primary outcomes</u>

The co-primary outcomes are mental health (compositive measure PHQ-ADS) and quality of life (ICECAP-A) at 12-months.

<u>Secondary outcomes</u>

- Patient Health Questionnaire Anxiety and Depression (PHQ-ADS), and ICEpop CAPability Measure for Adults (ICECAP-A), EuroQol Quality of Life (EQ-5D-5L)
- harmful substance use (Maudsley Addiction Profile (MAP), Leeds Dependence Questionnaire, (LDQ))
- risk taking behaviours (MAP)
- social functioning including occupation/education roles (MAP)
- physical health (MAP, EQ-5D-5L)
- housing status (self-report housing status)
- social outcomes, therapeutic alliance with the Peer Navigator (intervention group) and support workers (control group), and service accessibility (items from the Social Satisfaction Questionnaire (SSQ)
- service utilisation (MAP, self-report service utilisation (health, social care, and criminal justice), items from Client Evaluation of Self and Treatment (CEST))
- Relational empathy (Consultation and Relational Empathy measure (CARE))

Outcome measures will be assessed at baseline, and 6-, 12-, and 15-months post baseline allowing us to compare trajectories of outcomes during and after intervention between groups. The CARE

© University of Stirling

measure which asks participants about their relationship with their Peer Navigator (intervention condition) or Support Worker (control condition) will only be assessed at 6-, 12- and 15-months as participants will not have started working with the Peer Navigator at baseline. Excluding baseline data collection, a 12-week window (with six weeks either side) will be allowed for each data collection timepoint; given the characteristics of the target group we will seek to collect data wherever possible even if this is outside the 12-week window. The primary measurement point is 12 months post-baseline. The 15-month point has been included to gather information on whether there is any (rapid) post-intervention dissipation of intervention effects. This knowledge will be important for health/social care commissioners. Electronic data collection will be supported via a bespoke database with management tools designed by our Clinical Trials Unit CHaRT (University of Aberdeen). In the unlikely event of technical failure, the data will be collected using paper copies of the questionnaires.

Study Researchers, who are independent of intervention delivery, will collect outcomes from participants in face-to-face meetings where the database will be completed on study tablets by the researchers. Baseline demographic information will also be collected from participants including:

- participant age, gender, marital status, ethnicity, and disability status
- education
- Armed Forces experience and care system experiences
- medication use

Study researchers carrying out follow-up data collection at 6-, 12- and 15-months will also ask participants to update the contact information the research team hold about them and will complete a contact details form at each data collection timepoint.

11.2 Data transfer

All data will be stored in line with General Data Protection Regulation (GDPR). The University of Stirling are leading and will be responsible for all process evaluation data and data collected from the Peer Navigators and staff. Only the research team at the University of Stirling who are involved in the day-to-day conduct of the study will have access to participants' identifiable information stored at the University of Stirling trial office. Anonymised and pseudonymised research data will be stored in secure SharePoint site for the study. Paper copies of consent forms will be scanned and saved in the SharePoint site and hard copies will be stored securely on site at the University of Stirling. Any identifiable data accessed outwith the University of Stirling campus will be password protected and shared only via a secure online system (i.e., audio files and transcripts being transferred to and by the transcriber). The research team will use an external transcriber (TP Transcription) who has signed a data protection/confidentiality agreement with University of Stirling. After each interview, the researcher will upload the recording from the audio recorder to the secure SharePoint site and immediately delete the recording from the audio recorder. Once the recordings are uploaded, they will be transferred to TP Transcription for transcribing and once transcribed and checked/pseudonymised, they will be deleted by the research team from the SharePoint site. Pseudonymised research data (i.e., transcripts) will be stored in the secure SharePoint site. Transcripts and consent forms will be stored for 10 years after the study has ended.

We will hold client and service staff participant contact details (name, address, phone number and email address if they have one) to contact them for data collection and dissemination purposes. These details will be stored in a separate password-protected Excel spreadsheet on the SharePoint site along with their unique reference ID. All data collected will be stored in password protected spreadsheets on the secure SharePoint site. Names and contact details will be kept for 24 months after the study has ended. After this point these details will be deleted.

 © University of Stirling

The University of Aberdeen are leading and will be responsible for all client participant outcome measures data. These data will be collected as noted in the section above, on a bespoke data collection and management tool designed by the CHaRT research team. Data collection adheres to study specific operation manuals and SOPs. Once testing concludes, the CHaRT Senior IT manager or Senior Programmer issues a 'Database Authorisation to Go Live' form. This form signals the completion of testing, acceptance of validation sheets, and readiness for the database to go live. Intervention and control group participants will complete the outcome measures as noted in the sections above and these collected data will undergo pseudonymisation at the time of collection via an allocated participant ID number. No names or contact details will be recorded in the CHaRT data collection system.

As noted above, University of Stirling researchers will ensure that client participant names are logged on a separate spreadsheet (noted above) that is not connected to outcome measures data. This spreadsheet will have client participant names and contact details alongside their allocated ID number so that data collected across measure timepoints can be associated with that participant. Contact details are also needed because we aim to share study results with all participants. Copies of study results will be provided to all services and shared with intervention and control group participants.

Quantitative client participant outcome data will be hosted on the University of Aberdeen data centre. The University of Aberdeen provides and manages a resilient networked data storage solution which is replicated continuously to a disaster recovery site and backed up nightly. Strong network protection is provided in the form of isolation of services from each other and data stores. Access to the data is facilitated through a secure web application, the CHaRT data collection tool. Users, with pre-approved roles, need a username and password for entry, ensuring viewing or editing rights only for specific, location-based (centre) personal data. Access, being web-based, is location-independent. The website employs SSL/HTTPS with a minimum of 3DES SHA-1 encryption. Data will thus be linked to a pseudonymised ID number at University of Aberdeen, and all personal details will be stored securely at University of Stirling.

12. Safety

The interests of all participants, including Peer Navigators and The Salvation Army staff, will be guarded by normal duties of care, following appropriate information and clinical research governance approval procedures.

12.1 Participants

It is not expected that serious adverse events will result from taking part in the trial. However, given the vulnerability of people involved in the study, there will be a process for reporting and recording disclosures of current or future intent to harm themselves or others, or situations of actual / potential harm having taken place/at risk of taking place (this will be breaking confidentiality which is detailed on the participant information sheet and consent form for the study).

Most of the adverse events reported in the SHARPS study will be due to responses to a specific question in the PHQ-9 measure of depression which asks about suicidal thoughts or harm to self in the last 2 weeks. When this question is answered with any option other than ‘not at all’, the researcher is expected to inform staff in the service and complete an adverse events form for the participant. If such disclosures do occur, these will be shared by the researcher with service staff on the day of recruitment/data collection. Service staff will conduct a risk assessment as per their duty of care and The Salvation Army responsibilities. Researchers are not additionally required to verbally discuss such adverse events with the study leads unless they are seriously concerned about a participant’s wellbeing.

An adverse events form describing the disclosure and the action taken will be completed by the researcher (with only the participant identifier code being recorded on the form) via the CHaRT data collection tool. These will all be notified electronically via the CHaRT website to the Study lead, Deputy Study Lead and Cluster Lead (Tessa Parkes/Hannah Carver/Jen Boyd). They will also be notified by the researcher taking the disclosure to the Study Leads/Cluster Leads (above) via email. At the end of the recruitment/data collection day in service, the designated lead for adverse events (usually Hannah Carver but also Tessa Parkes/Jen Boyd as needed) will go onto the study website and sign off on each of the adverse events from that day, checking that actions taken by researchers are appropriate.

At the end of each recruitment/data collection day in a service, the researchers will report the numbers of participants recruited to the study/who have done data collection to the cluster lead (Hannah Carver/Jen Boyd) who will then summarise the numbers to the Service Manager/Programme Manager. Any adverse events will be included in this email. The email will note the initials of the participants where there was an adverse event and what staff members were told about this (in line with the above) at the time. The participant initials and year of birth for individuals who have experienced an adverse event related to their response to the PHQ-9 in control service sites will also be reported The Salvation Army Senior Management in order for them to take forward safeguarding processes with control site service managers. There will be no other "more traditional" safety reporting of other adverse events within SHARPS.

Given that some participants may no longer be supported by The Salvation Army at 6-, 12- and 15-month follow up points, researchers will follow a different adverse events process for these individuals. If the participant reports thoughts of suicide/self-harm in responde to the PHQ-9 or other issues, the data collector will remind them that they will have to inform the Study/Cluster leads about the disclosure. The researcher will also provide the participant with information about national/local support resources, including suicide helplines, via an information sheet at the end of the data collection session. The researcher will also tell the participant that a member of the study team will contact them (via phone, text and/or email) within 3 working days. If the data collector believes that the participant is in immediate danger, they will call the Police on 999. The data collector will then complete the usual adverse events form describing the disclosure and the action taken (with only the participant identifier code being recorded on the form) via the CHaRT data collection tool. An identified member of the study team will then contact participants within 3 working days to check in with them, ask them how they are feeling, and remind them of relevant services for support or to contact their GP. Three attempts will be made to contact the participant, if there is no response this will be recorded. The study team member will then log the participant contact and details of the call on a follow up risk assessment log stored on the SharePoint site. Co-investigators with specialist expertise in mental health will review the actions taken for all participants.

Any deaths will be recorded through the Change of Status, including cause and date of death where known.

12.2 Research staff and Peer Navigators

We will take all reasonable steps to ensure staff safety. Researchers will undertake training including GCP training provided by NIHR, follow clear safety protocols, and should disclose any adverse events (e.g., violence and intimidation) they experience to the study leads and an adverse event form should be completed. All adverse event forms will be entered into the CHaRT data collection tool and stored electronically on the secure SharePoint. Risk assessments will also be conducted for the researchers throughout the study using university procedures. Any issues will be discussed with a member of the study team (Parkes, Carver, Boyd). Given the vulnerability of participants in the study, researchers will also be provided with additional training around suicide and reviewed, with additional support provided where required.

Peer Navigators will be provided with support and training around safety. Peer Navigators will be under the line management of The Salvation Army staff and will follow The Salvation Army procedures around adverse events and safeguarding reporting when working within the service. However, given that the role will involve outreach support to clients Peer Navigators should disclose any adverse events or safeguarding concerns they experience (including stalking, violence, intimidation, witnessing a crime, relapse) to their line manager within The Salvation Army immediately as soon as an incident occurs, contact the study leads and an adverse event form should be completed. Peer Navigators will also be encouraged to discuss issues arising from their role (e.g., clients encroaching on or testing boundaries, their own risk of relapse) in line management and clinical supervision. A lone working policy will be in place for Peer Navigators and lone working apps for phones will be provided where this is desirable for the Service Manager/Peer Navigator to have. Research staff will also have a lone working policy and risk assessment in place.

13. Statistical, health economic and process evaluation outcomes

13.1 Primary outcomes

Participant mental health (PHQ-ADS) and quality of life (ICECAP-A) at 12 months post baseline assessment.

13.2 Secondary outcomes

Trial participants

Primary outcomes at collected 6- and 15-months post baseline assessment:

- Mental health (PHQ-ADS)
- Quality of life (ICECAP-A)

Secondary outcomes collected at 6, 12 and 15-months post baseline assessment:

- Harmful substance use (MAP, LDQ)
- Risk taking behaviours (MAP)
- Social functioning (MAP)
- Physical health (MAP, EQ-5D-5L)
- Housing status (self-reported)
- Social outcomes, therapeutic alliance with the Peer Navigator (intervention group) and support workers (control group), and service accessibility (items from the SSQ)
- Service utilisation (MAP, self-report service utilisations, items from CEST)
- Relational empathy (CARE)

Peer Navigators and intervention service staff

Collected at 6- and 12-months post baseline assessment:

- Normalisation Measure Development Questionnaire (NoMad)

Peer Navigators & control site Support Workers

Collected at 12 months post baseline assessment:

- Professional Quality of Life Scale (ProQoL)

- Job Satisfaction Survey (JSS)

Safety

Collected throughout the trial:

- Number and proportion of adverse events
- Number of adverse events per participant
- Details of adverse events including seriousness

Economic evaluation

Collected at 6-, 12- and 15-months post baseline assessment:

- Quality of life (EQ-5D-5L and ICECAP-A)
- Use of NHS, local government, third sector and criminal justice services

The Salvation Army Service observations

Observations will be undertaken by Researchers attending services when collecting other outcome data across both arms to track the context of The Salvation Army services at baseline, 6-, 12- and 15-month follow-ups.

14. Statistical considerations

14.1 Sample size

The unit of randomisation is city/town (the clusters), each cluster will recruit 25 participants (based on maximum possible caseload for each Peer Navigator) in the intervention arm and 30 participants in the control arm. A trial of 20 clusters will recruit 550 participants: we anticipate an attrition rate of up to 40% in the intervention arm and 50% in the control arm, based on feasibility work/related research resulting in outcome data on 300 participants (150 in each arm), equating to mean cluster size of 15. Assuming an ICC of 0.01 this design has 90% power to detect a 0.4SDs effect size. We will work our PPI collaborators to maximise retention in the study, refining processes as we go.

As part of the process evaluation, interviews will be undertaken with staff in the intervention services and with all the 10 Peer Navigators, to understand experiences of, and views on, the intervention from a range of perspectives, and to collect data on changes in the trial contexts during the study:

- Staff in a range of roles in intervention clusters (n=10)
- External stakeholders, including commissioning roles in intervention clusters (n=10)
- Staff in a range of roles in control clusters (n=10)
- Peer Navigators (n=20, i.e., all 10 Peer Navigators at two time points, pre and post intervention)

We will conduct the NoMad questionnaire [40] with a sample of staff (n=4-5) in each intervention setting at three time points (start, middle and end of intervention, aiming for a total sample size of 100-120).

14.2 Frequency of analysis

There will be only one final analysis of all outcome data, conducted after the trial is closed to recruitment and the final 12- and 15-month follow-up data have been received. Analyses will only be carried out when all outcome data have been received and the database for follow-up data have been cleaned and locked.

Interim reports will be presented to the Trial Steering Committee and the Data Monitoring and Ethics Committee, containing descriptive information annually (or when requested by the committees). Reports will contain information on recruitment, data collection, safety, and data quality.

14.3 Primary outcome analysis

Statistical analysis will be conducted according to a detailed statistical analysis plan which will be finalised prior to the start of data collection. Baseline and outcome data will be described using summary statistics, broken down by group. All analyses will be based on the intention-to-treat principle. Primary outcomes will be analysed using a repeated measures mixed effects linear model extended for cluster randomised trials to include a random effect for cluster and as well as participant [43]. Models will include a fixed effect for treatment, nominal time, country (Scotland/England), and the baseline outcome score. Treatment effects will be estimated at each time point using a treatment-by-time interaction: the primary measurement time point is 12 months after recruitment into the trial. A small sample approximation will be applied to the degrees of freedom given the number of clusters [44].

14.4 Secondary outcome analysis

Secondary outcomes will be analysed in a similar way, with generalised linear models appropriate for the distribution of the outcome. All treatment effects will be presented using 95% confidence intervals. We plan no adjustments for multiple outcomes, nor any interim efficacy analysis, only one final analysis.

14.5 Interim analyses

There are no planned interim analyses, other than the data required for review by the Data Monitoring and Ethics Committee. It is not anticipated that this trial will be stopped early for efficacy or safety reasons.

14.6 Handling missing data

The primary analysis will use an unstructured time and covariance structure, which gives unbiased treatment effects when outcome data are missing at random (MAR). A MAR mechanism is unlikely to be the case in this population, and we will explore the impact of missing data using pattern mixture models under missing not random assumptions using models for repeated measures data in cluster randomised trials outlined by Fiero et al. [45].

14.7 Success and progression criteria

We have set two targets: one to assess the opening of the clusters as recruitment sites, and the other the recruitment of participants. We propose one decision point at month 10 of the study, to be assessed by the Trial Steering Committee, and our progression criteria are laid out in Table 3 below. We have built contingency into the timetable to take account of unpredictable service demands. There is no internal pilot phase given the short period of recruitment of both clusters and participants.

Table 2: Stop/go criteria at 10 months

	GREEN	AMBER	RED
**Cluster opening**	100% (20 clusters)	70-95% (14–19 clusters)	< 70% (<14 clusters)

 © University of Stirling

Participant recruitment*	100% (12.5 per cluster month)	60-96% (7.5–12 per cluster month)	< 60% (<7.5 per cluster month)

** For participants, stop/go will be based on expected number of participants recruited given the number of clusters open to recruitment, to distinguish between cluster- or participant-level barriers to recruitment.*

Figure 3 shows the recruitment of clusters and participants over the four-month recruitment phase. Our projection is based upon 20 clusters contributing 25 or 30 participants each over two months. Incorporating a staggered cluster set-up, we would expect to recruit 65 participants in month 7, 500 by the end of month 9, and the remaining 50 in month 10. Recruitment is based upon throughput from our previous feasibility study, use of study-specific researchers to recruit, and having existing 'pools' of participants in the cluster services.

Figure 3: Recruitment projection

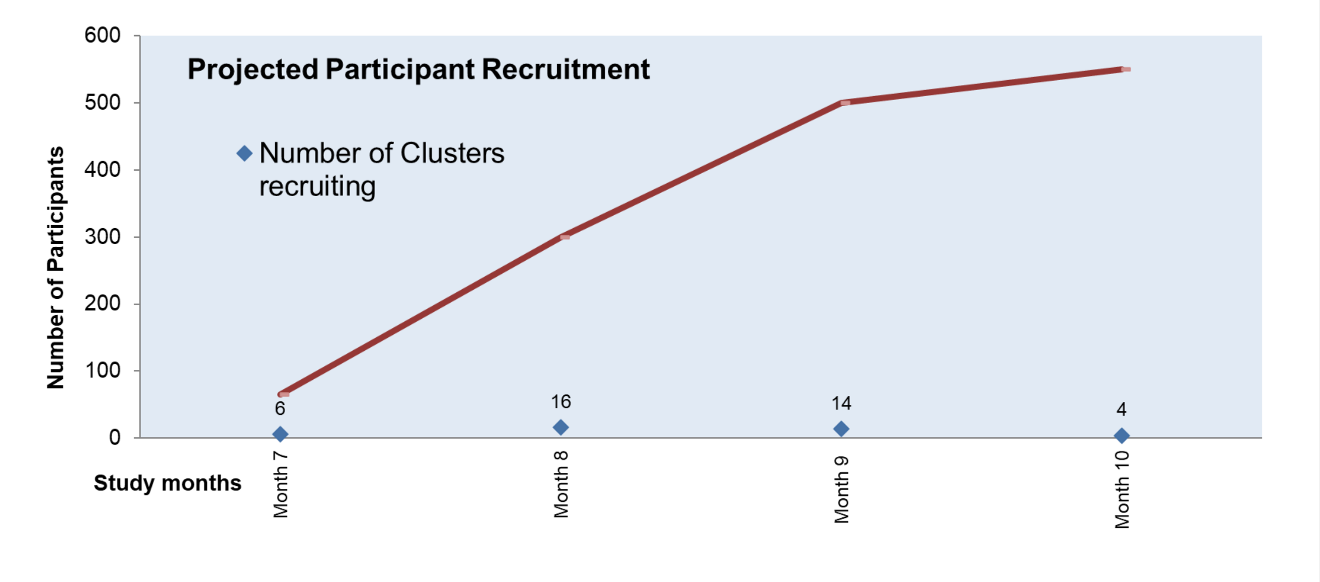

To determine success criteria and barriers, we have a risk/mitigation plan with a selection of issues below.

Table 3: Risks and mitigations

Potential risk	How we intend to mitigate risk
Study sites not meeting recruitment milestones	Support of all sites has been secured by The Salvation Army. If there are problems with a particular site, we have resource for extra support to increase recruitment.
Difficulties recruiting Peer Navigators	We will use learning from the SHARPS feasibility study and work closely with The Salvation Army and our networks to recruit the Peer Navigators. If it is not possible to recruit a Peer Navigator in a cluster (after two attempts of advertising the post) then the cluster will be swapped out of the study and replaced with one of several The Salvation Army clusters that were held as 'back-pocket' clusters if needed. The decision on which cluster should act as the

	replacement will be decided based upon the optimal value balance statistics and be agreed with NIHR and our study DMEC.
Peer Navigator(s) leave role early/experiences crises	We will use our learning from the SHARPS feasibility study and either recruit a new Peer Navigator or identify an alternative solution in response to Peer Navigator challenges, depending on the circumstances and/or when they leave.
Data collection issues	Three job share Trial Managers are involved, alongside an experienced (G8) Research Fellow, a (G6) part time Research Assistant who is supporting the process evaluation, and eight Research Assistants to ensure data are collected appropriately, with active support/management from staff within The Salvation Army.
COVID-19 restrictions affecting trial	We will put in place mitigations if there are any COVID-19 restrictions that come into place during the trial. This is currently hard to plan for in advance.
Decommissioning of all The Salvation Army services in one cluster	If all of The Salvation Army services within one of the clusters are decommissioned during the 12-month intervention period, we will put in place the following mitigations:<br>1) Intervention settings: the Peer Navigator will continue to deliver the intervention to participants in settings outside of The Salvation Army services and the line management of the Peer Navigator will be transferred to the study lead at the University of Stirling<br>2) Control settings: Research Assistants will attempt to follow up control participants and collect data from them in settings outside of The Salvation Army services in public locations such as other homelessness and substance use services in the area

15. Health economic evaluation

A full economic evaluation will be conducted from a public sector perspective. This will take the form of a cost-utility analysis. We will also conduct a cost-consequence analysis which will identify, and where possible measure, all costs, and consequences (effects) of the intervention, compared to control. Given that the intended effects of the intervention are wider than health effects, we will estimate both an incremental cost per quality-adjusted life year (QALY) gained and an incremental cost per Years of Full Capability (YFC) gained. Capability is measured using the ICECAP-A measure which measures broader wellbeing.

The direct intervention costs include the salary costs of the Peer Navigators, and costs borne by The Salvation Army. Public sector perspective costs will include costs of health and social care (primary care, secondary care, community care, medication), criminal justice services, and housing services. Resource use estimates will be combined with unit costs obtained from standard sources or study specific estimates. Total costs will be reported, as well as total costs by organisation. Incremental costs for the SHARPS intervention versus control will be estimated using mixed effects generalised linear models, with appropriate distributions for cost data and adjustment for baseline covariates.

We will measure benefits in terms of QALYs gained, based on participant responses to the generic EQ-5D-5L health-related quality of life measure [46], and in terms of YFC, based on participant responses to the ICECAP-A capability measure [47]. Both are measured at baseline, 6, 12 and 15

months. The EQ-5D-5L and ICECAP-A responses will be converted into utility scores using published population tariffs. Incremental QALYs and YFCs for the SHARPS intervention versus control will be estimated using mixed effects generalised linear models, with adjustment for baseline covariates.

Costs and benefits will be combined to estimate incremental cost per QALY gained and incremental cost per YFC gained for the SHARPS intervention versus control over the trial follow up period. Deterministic sensitivity analyses will be undertaken to test the impact of assumptions and analysis methods on results. A comprehensive set of sensitivity analyses, including exploration of appropriate missing data models will be undertaken to explore uncertainty in our conclusions. Results will be plotted on the cost-effectiveness planes to illustrate the impact of sampling uncertainty on results. The cost consequence analysis will be presented as a balance sheet listing all relevant costs and effects.

16. Process evaluation

In terms of methodological theory, within our SHARPS feasibility study, NPT guided the development/implementation of the intervention, as well as the process evaluation. NPT comprises four components: coherence (understanding), cognitive participation (buy-in), collective action (making it work), and reflexive monitoring (on-going appraisal). For the SHARPS cRCT we will draw on May et al.'s [48] coding manual to facilitate transparent data analysis processes and reduce the cognitive and practical burden on researchers. This manual also links the 12 primary NPT constructs to realist evaluation methods by conforming to the Contexts-Mechanisms-Outcomes configuration that realist approaches centre upon. We will conceptually draw upon realist frameworks. To interpret findings, we will also use a range of theories/frameworks, such as the modified access model by Penchansky and Thomas [49], and on peer support, such as Barker et al.'s (2020) model of change mechanisms within unidirectional peer support [50]. We will examine the impact on the Peer Navigators using literature on task shifting, organisational cultures, social bonds, and intersectionality.

The analysis will identify contextual influences on implementation across settings: how individuals understood, adopted, or perceived the intervention; how participants engaged with/disengaged from the intervention; how staff experienced hosting the intervention and being in the control (standard care) settings; how the Peer Navigators made sense of role; and other contextual factors impacting delivery. Analysis will be undertaken using the Framework approach and NVivo software will be used to organise and code data to support the process of analysis. All stages of Framework will be closely followed. To enhance rigor and validity, the trial EbyE group will participate in data analysis/interpretation to act as a form of 'member checking' to enhance the validity and trustworthiness of the findings. As part of the process evaluation, we will also take a mixed methods approach to assess intervention fidelity, this will include the use of an adapted existing fidelity index.

17. Experts by Experience group

Our EbyE group will be closely involved in the research process throughout the trial. This includes co-producing all participant materials to ensure they are easy to understand, participating in data analysis and interpretation to act as a form of 'member checking' to enhance the validity and trustworthiness of the findings, and collaborating to produce study outputs. The EbyE group will meet every six months throughout the trial chaired by Steele and Wallace and will comprise of individuals with lived/living experience of homelessness and/or substance use (and related challenges), including some of those involved in the SHARPS feasibility study.

 © University of Stirling

18. Data monitoring

18.1 Source data

The study is set-up such that quantitative data can be captured electronically directly into the study database. We anticipate that the majority of quantitative data will be captured in this way, and therefore the source document is the electronic record. Researchers will have access to paper copies of data collection tools as a failsafe if, for example, there is no wi-fi or 4G internet access. If paper copies of data collection tools are used, these will be considered to be the source document and retained in the study record. Any paper copies will be returned to the University of Stirling, scanned and uploaded to the SharePoint site.

18.2 Data monitoring

Data collected by Researchers will be reviewed by the trial office staff to identify data queries and/or missing data. Data queries will be raised to try and ensure a complete and accurate data set. Extensive range and consistency checks will further enhance the quality of the data. Cleaning will take place ahead of each Data Monitoring and Ethics Committee meeting.

18.3 Clinical governance issues

To ensure responsibility and accountability for the overall quality of care received by participants during the trial period, clinical governance issues pertaining to all aspects of routine management will be brought to the attention of the Trial Steering Committee and, where applicable, to local The Salvation Army services.

19. Quality assurance and ethical considerations

19.1 Quality assurance

The trial will be conducted in accordance with the MRC Good Clinical Practice guidance and the Research Governance Framework for Health and Social Care. Universities (Aberdeen/Stirling) have codes of practice for secure data management, researcher conduct and safety.

19.2 Ethical considerations

The main ethical considerations are ensuring use of appropriate lay language, confidentiality/anonymity, data protection, participant burden, disclosure of risk, safety of researchers, safety of Peer Navigators, and dealing with the end of the intervention in a sensitive manner. Our EbyE group will ensure that all study materials are written using lay language. All research staff and Peer Navigators will sign a form stating confidentiality/anonymity will be adhered to. Participants may feel the burden of completing the measures at several time points (as well as the potential to also be interviewed), particularly those in the standard care sites. We will provide refreshments and vouchers to those completing the outcome measures and vouchers and refreshments to interview participants; study requirements will be clearly explained to all potential participants. We will comply with HRA Guidance for Informed Consent and GDPR. Participants will be asked to state clearly that they accept and understand limitations to confidentiality within the qualitative interviews. These limitations refer to disclosures of current or future intent to harm themselves or others.

If such disclosures do occur, these will be shared with service staff who will conduct a risk assessment as per their duty of care. The individual who has taken the disclosure would discuss course of action with study leads and an adverse events form completed. Staff safety will be ensured, with researchers following clear safety protocols, and risk assessments will be conducted,

and Peer Navigators provided with support/training around safety. Finally, we will use the learning from the SHARPS feasibility study regarding ending the intervention to ensure that transition support is in place. Ethical approval for this trial has been granted by University of Stirling (Ref: NICR 2024 16751) and The Ethics Subgroup of the Research Coordinating Council of The Salvation Army.

19.3 Study amendments

Substantial amendments to the study (such as reduction of outcome measures) should be discussed by the Project Management Group, and where appropriate the Trial Steering Committee and Data Monitoring and Ethics Committee. Amendments to the protocol are reviewed by the funder after ethics approvals are sought.

19.4 Urgent safety measures and serious breaches of good clinical practice

The study lead and research staff may take immediate safety measures to protect research participants against any hazard to their health or safety without prior authorisation from the research ethics committees or sponsor. However, they must alert the sponsor as soon as possible.

In the event that a serious breach of good clinical practice (GCP) is suspected, this will be reported to the sponsor and two ethics committees immediately and will be investigated by the sponsor. Any corrective action required will be undertaken by the study lead and ethics committees will be informed. If necessary, a protocol amendment will be submitted for review.

19.5 Satellite studies

It is recognised, that the value of the trial may be enhanced by smaller ancillary studies of specific aspects. Plans for these will be discussed in advanced with the project management group and, if appropriate, with the Trial Steering Committee. Depending on the nature of the satellite trial, the Sponsor may consider this to be an amendment to the ethical approval for the study, or to require ethical approval as a project in its own right.

20. Confidentiality

All information shared within a data collection context will be kept confidential unless there are reasons to breach confidentiality. In addition, participants will be asked to clearly state that they accept and understand limitations to confidentiality within interviews/data collection sessions. These limitations refer to disclosures of current, potential, or future intent to harm themselves or others, or harm to themselves from other parties. The disclosure from the participant must include clear indication/intent of current (active) or future threats of significant harm towards a specified person or themselves. Significant harm includes but is not limited to self-harm, suicidal thoughts, the use of weapons, sexual or physical violence, and general safeguarding concerns for children and vulnerable adults. If such disclosures do occur, this information will be shared with Study Leads, Service Managers and/or The Salvation Army study senior leads as appropriate who will take actions as needed according to organisational policies and procedures.

All data will be anonymised or pseudonymised, and all information that may be used to identify participants will be altered or removed. Each participant will be allocated a unique identifier study code which will be detailed on their consent form. Data will be collected, stored, and accessed in accordance with GDPR. The study team has a data protection/confidentiality agreement with the external transcriber. The audio files will be deleted once they have been transcribed/checked. To protect the identity of study participants, no names will be used in the reporting of the study. We will instead use numerical IDs followed by generic role descriptors such as ‘staff’ and ‘external stakeholder’.

21. End of trial and archiving

21.1 Definition of end of trial

The end of follow-up for each participant is defined as completion of the follow-up visit at 15 months. The end of follow-up is when the last participant completes their follow-up visit at 15 months. The end of the trial is defined as the end of funding (31st March 2027).

The Funder, Sponsor, study team and/or the Trial Steering Committee have the right at any time to terminate the study for administrative or other reasons.

If terminated prematurely, the study team will inform participants and ensure that the appropriate follow up is arranged for all involved.

An end of trial report will also be issued to the funders at the end of funding.

21.2 Archiving

All study documentation will be kept for at least 10 years from the end of the study. Researchers obtaining consent will return consent forms and completed eligibility screening forms (ASSIST-Lite screening tool) to the SHARPS trial office in Stirling where they will be securely stored. If a potential participant did not meet the inclusion criteria (they score below the threshold on the ASSIST-Lite screening tool), all documentation will be shredded on The Salvation Army service premises and not returned to the trial office in Stirling. If electronic data collection is not possible, at the end of each participant's follow-up, Research Assistants will collect the data using paper copies of the questionnaires. Research Assistants are required to input any data they collect using paper copies into the CHaRT data collection tool as soon as the technical issue is resolved. Research Assistants will store any paper copies of anonymised participant data until they have finished collecting the participant data at that timepoint. A courier or tracked delivery will then be arranged to return any paper copies to the University of Stirling for safe storage. Adverse event forms and change of status forms will be completed and returned via email to the study leads at the University of Stirling (Parkes and Carver for adverse events and Boyd for change of status), or be entered electronically directly onto the SHARPS website database (where the University of Stirling team will be automatically notified by email). Research Assistants and Peer Navigators completing adverse event forms or change of status forms will also inform the study lead (Parkes) or Deputy Study Lead (Carver) by phone call that a form has been completed and returned, if there is no response by email after 12 hours. If not captured electronically, once received, all adverse event forms and change of status forms will be recorded in the CHaRT tool by research staff at the University of Stirling. Where appropriate, the study leads at the University of Stirling will follow up with those reporting the incident to ensure that further action has been taken to ensure the safety of those involved. There will be no files stored at the trial sites, all files will either be stored on the SharePoint system, or securely within the University of Stirling or the University of Aberdeen.

Data will be archived by CHaRT for at least 10 years after the end of the study.

22. Statement of indemnity

The trial sponsor is the University of Stirling, and the University of Stirling will be liable for negligent harm caused to participants arising from the management of the research.

23. Trial organisational structure

23.1 Responsibilities

Sponsor

University of Stirling will sponsor the study and therefore has the responsibility for confirming there are proper arrangements to initiate, manage, monitor, and finance the trial.

Project Management Group

The strategic oversight of the trial will be provided by a Project Management Group, consisting of the grantholders (Chief Investigators and all other co-investigators), the Trial Managers (Stirling/Aberdeen), the Research Fellow (Stirling), Research Administrator, statistician and health economists and other senior members of the Trials Unit, and a representative from the study partner (The Salvation Army).

Trial Management Group

The management of the trial will be coordinated by a Trial Management Group consisting of the grantholders (Chief Investigators and a smaller group of co-investigators as required), the Trial Managers (Stirling/Aberdeen), the Research Fellow (Stirling), Research Administrator (Stirling), statistician, health economists and other senior members of the Trials Unit. This group will be responsible for making decisions concerning ongoing management of the trial and deal with any problems as they arise.

Core Trial Group

The day-to-day running of the trial will be the responsibility of the Core Trial Group which will consist of the Chief Investigators (Stirling/Aberdeen), the Trial Managers (Stirling/Aberdeen), Research Fellow (Stirling), Research Administrator (Stirling), and statistician.

24. Trial oversight

24.1 Independent Trial Steering Committee

A Trial Steering Committee (TSC), with independent members, will be established to oversee the conduct and progress of the trial. The membership and terms of reference of the Trial Steering Committee will be filed in the trial master file.

24.2 Data Monitoring and Ethics Committee

An independent Data Monitoring and Ethics Committee (DMEC) will be established to oversee the safety of subjects in the trial. The membership and terms of reference of the Data Monitoring and Ethics Committee will be filed in the trial master file.

25. Dissemination

Ownership of the data arising from this study resides with the study team and their respective employers. On completion of the study, the study data will be analysed, and a study report will be prepared. Authors will acknowledge that the study was funded by the NIHR HTA, and other contributors will be acknowledged. The study report will be used for publication and presentation at scientific meetings. Investigators have the right to publish orally or in writing the results of the study.

To safeguard the integrity of the main trial, reports of explanatory or satellite studies will not be submitted for publication without prior arrangement from the Project Management Group and Trial Steering Committee.

Once the main trial findings have been published, a lay summary of the findings will be sent to participants.

More detailed plans for this dissemination will be considered and developed with input from the EbyE group through the duration of the trial and will be finalised as part of the close-out plans.

26. Publication Policy

1. DEFINING AUTHORSHIP

Authorship of published or presented papers is based on the following criteria.[1]

i. Substantial contributions to the conception or design of the work; or the acquisition, analysis, or interpretation of data for the work; AND

ii. Drafting the work or revising it critically for important intellectual content; AND

iii. Final approval of the version to be published; AND

iv. Agreement to be accountable for all aspects of the work in ensuring that questions related to the accuracy or integrity of any part of the work are appropriately investigated and resolved.

In addition to being accountable for the parts of the work he/she/they has done, an author should be able to identify which co-authors are responsible for specific other parts of the work. In addition, authors should have confidence in the integrity of the contributions of their co-author.

2. PRINCIPLES OF AUTHORSHIP

The following principles of authorship have been derived from editorial publications from leading journals[2,3] and are in accordance with the rules of the International Committee of Medical Journal Editors (ICMJE)[1].

All contributors must fulfil the criteria detailed in section 1: DEFINING AUTHORSHIP in order to qualify for authorship.

Contributors who meet fewer than all four of the criteria for authorship listed above should not be listed as authors, but they should be acknowledged. For example, participation solely in the acquisition of funding, collection of data or technical editing, language editing or proofreading the article is insufficient by itself to justify authorship[1]. Those persons may be acknowledged and their contribution described. See section 3: ACKNOWLEDGEMENTS.

a. Preferred CHaRT authorship

Where possible, all CHaRT trials should publish using all the named contributors who qualify for authorship in the byline i.e. Jane Doe, John Doe, John Smith and Ann Other.

However, there may be situations where this is not possible, for example if the journal limits the number of authors. In such circumstance, group authorship may be appropriate using bylines similar to “The XXXXX trial group” or “Jane Doe, John Doe, John Smith, Ann Other and the XXXX trial group”. The article should carry a footnote of the names of the people (and their institutions) represented by the corporate title. For some journals the journal will provide instructions on how to ensure the names of the collaborators appear on PubMed or equivalent.

Group authorship may also be appropriate for publications where one or more authors take responsibility for a group, in which case the other group members are not authors but may be listed in the acknowledgement (the byline would read 'Jane Doe for the Trial Group') 2. Again, the article should carry a footnote of the names of the people (and their institutions) represented by the corporate title.

b. Determining authorship

These authorship criteria are intended to reserve the status of authorship for those who deserve credit and can take responsibility for the work. The criteria are not intended for use as a means to disqualify colleagues from authorship who otherwise meet authorship criteria by denying them the opportunity to meet criterion numbers (ii) or (iii). Therefore, all individuals who meet the first criterion should have the opportunity to participate in the review, drafting, and final approval of the manuscript1.

Tentative decisions on authorship should be made as early as possible3. These should be justified to, and agreed by, the Project Management Group (PMG). Any difficulties or disagreements will be resolved by the Trial Steering Committee (TSC).

c. Ordering of authors

The following rules may help with the ordering of authors, particularly for publications with individual authorship:

i. The person who has taken the lead in writing may be the first author.
ii. The senior author may wish to be the last named author.
iii. Those who have made a major contribution to analysis or writing (i.e. have done more than commenting in detail on successive drafts) may follow the first author immediately; where there is a clear difference in the size of these contributions, this should be reflected in the order of these authors.
iv. All others who fulfil the four authorship criteria described in Section 1: DEFINING AUTHORSHIP may complete the list in alphabetical order of their surnames.

3. ACKNOWLEDGEMENTS

All those who make a contribution to a publication, but who do not fulfil the criteria for authorship, such as interviewers, data processors, staff at the recruiting sites, secretaries and funding bodies, should be acknowledged by name, usually in an 'Acknowledgements' section specifying their contributions. Because acknowledgment may imply endorsement by acknowledged individuals of a trial's data and conclusions, authors are advised to obtain written permission to be acknowledged from all acknowledged individuals[1].

The acknowledgements should also reflect any agreed acknowledgements (for example with suppliers) that were documented in supply agreements (or equivalent).

4. DISCLAIMERS

All papers arising from CHaRT must include the full title of the Health Services Research Unit (HSRU) and the appropriate disclaimer specified by the Chief Scientist Office (CSO). For the current disclaimer please see Q-Pulse.

Authors should also ensure they include the trial funder's disclaimer: refer to the funders website for details. Be aware that other disclaimers may also be required.

5. QUALITY ASSURANCE

Ensuring quality assurance is essential to the good name of the trial group. All reports of work arising from the SHARPS trial, including conference abstracts, outputs describing methodological aspects of the trial, and any outputs describing results from the trial, should be peer reviewed by the PMG. The PMG will be responsible for decisions about submission following internal peer review. Submission may be delayed or vetoed if there are serious concerns about the scientific quality of the report. If individual members of the group are dissatisfied by decisions, the matter may be referred to the TSC.

It is hoped that the adoption and dissemination of this policy will prevent disputes that cannot be resolved by informal discussion. However, any member of the trial team with a concern about authorship should discuss it with the relevant Chief Investigator, TSC, Line Manager or Programme Director as appropriate.

REFERENCES

1. Recommendations for the Conduct, Reporting, Editing, and Publication of Scholarly Work in Medical Journals. Developed by members of the ICMJE, the document is revised regularly and the current version (updated May 2022) is available at (www.icmje.org/#authors)
2. Huth EJ (1986). Guidelines on authorship of medical papers. *Annals of Internal Medicine*, **104**, 269-274.
3. Glass RM (1992). New information for authors and readers. Group authorship, acknowledgements and rejected manuscripts. *Journal of the American Medical Association*, **268**, 99.

References

[1] Liu M, Hwang SW. Health care for homeless people. Nature Reviews 2021. https://doi.org/10.1038/s41572-020-00241-2.

[2] Fornaro M, Dragioti E, De Prisco M, Billeci M, Mondin AM, Calati R, et al. Homelessness and health-related outcomes: an umbrella review of observational studies and randomized controlled trials. BMC Medicine 2022 20:1 2022;20:1–19. https://doi.org/10.1186/S12916-022-02423-Z.

[3] Amore K, Baker M, Howden-Chapman P. The ETHOS Definition and Classification of Homelessness: An Analysis. Eur J Homelessness 2011;5.

[4] The Scottish Government. The extent of homelessness in Scotland - 2022-23 2023. https://www.gov.scot/publications/homelessness-in-scotland-2022-23/pages/the-extent-of-homelessness-in-scotland/ (accessed January 29, 2024).

[5] Shelter. At least 271,000 people are homeless in England today - Shelter England 2023. https://england.shelter.org.uk/media/press_release/at_least_271000_people_are_homeless_in_england_today (accessed January 29, 2024).

[6] Hewett N, Halligan A. Homelessness is a healthcare issue. J R Soc Med 2010;103:306–7. https://doi.org/10.1258/JRSM.2010.10K028.

[7] Thomas B. Homelessness: A Silent Killer A research briefing on mortality amongst homeless people. London: Crisis 2011. https://www.crisis.org.uk/ending-homelessness/homelessness-knowledge-hub/health-and-wellbeing/homelessness-a-silent-killer-2011/ (accessed January 29, 2024).

 © University of Stirling

[8] Parkes T, Matheson C, Carver H, Foster R, Budd J, Liddell D, et al. Assessing the feasibility, acceptability and accessibility of a peer-delivered intervention to reduce harm and improve the well-being of people who experience homelessness with problem substance use: the SHARPS study. Health Technol Assess 2020;26:1–128. https://doi.org/10.1186/s12954-021-00582-5.

[9] Miler JA, Carver H, Masterton W, Parkes T, Maden M, Jones L, et al. What treatment and services are effective for people who are homeless and use drugs? A systematic 'review of reviews.' PLoS One 2021;16:e0254729. https://doi.org/10.1371/JOURNAL.PONE.0254729.

[10] Lowrie R, Stock K, Lucey S, Knapp M, Williamson A, Montgomery M, et al. Pharmacist led homeless outreach engagement and non-medical independent prescribing (Rx) (PHOENIx) intervention for people experiencing homelessness: a non- randomised feasibility study. Int J Equity Health 2021;20:1–13. https://doi.org/10.1186/S12939-020-01337-7/TABLES/5.

[11] McQueenie R, Ellis DA, McConnachie A, Wilson P, Williamson AE. Morbidity, mortality and missed appointments in healthcare: A national retrospective data linkage study. BMC Med 2019;17:1–9. https://doi.org/10.1186/S12916-018-1234-0/TABLES/2.

[12] Carver H, Ring N, Miler J, Parkes T. What constitutes effective problematic substance use treatment from the perspective of people who are homeless? A systematic review and meta-ethnography. Harm Reduct J 2020;17:1–22. https://doi.org/10.1186/S12954-020-0356-9/FIGURES/2.

[13] Harm Reduction International. What is Harm Reduction? 2024.

[14] Miler JA, Carver H, Foster R, Parkes T. Provision of peer support at the intersection of homelessness and problem substance use services: A systematic "state of the art" review. BMC Public Health 2020;20:1–18. https://doi.org/10.1186/S12889-020-8407-4/TABLES/4.

[15] Flike K, Aronowitz T. Factors That Influence Quality of Life in People Experiencing Homelessness: A Systematic Mixed Studies Review. J Am Psychiatr Nurses Assoc 2021;28:128–53. https://doi.org/10.1177/1078390320985286.

[16] Barker SL, Maguire · Nick. Experts by Experience: Peer Support and its Use with the Homeless. Community Ment Health J 2017;53:598–612. https://doi.org/10.1007/s10597-017-0102-2.

[17] Magwood O, Leki VY, Kpade V, Saad A, Alkhateeb Q, Gebremeskel A, et al. Common trust and personal safety issues: A systematic review on the acceptability of health and social interventions for persons with lived experience of homelessness. PLoS One 2019. https://doi.org/10.1371/journal.pone.0226306.

[18] Paisi M, Crombag N, Burns L, Bogaerts A, Withers L, Bates L, et al. Barriers and facilitators to hepatitis C screening and treatment for people with lived experience of homelessness: A mixed-methods systematic review. Health Expectations 2022;25:48–60. https://doi.org/10.1111/HEX.13400.

[19] Siersbaek R, Ford JA, Burke S, Cheallaigh CN, Thomas S. Contexts and mechanisms that promote access to healthcare for populations experiencing homelessness: a realist review. BMJ Open 2021;11:43091. https://doi.org/10.1136/bmjopen-2020-043091.

[20] Mercer F, Miler JA, Pauly B, Carver H, Hnízdilová K, Foster R, et al. Peer support and overdose prevention responses: A systematic 'state-of-the-art' review. Int J Environ Res Public Health 2021;18:12073. https://doi.org/10.3390/IJERPH182212073/S1.

[21] Baral S, Bond A, Boozary A, Bruketa E, Elmi N, Freiheit D, et al. Seeking shelter: homelessness and COVID-19. FACETS 2021;6:925–58. https://doi.org/10.1139/FACETS-2021-0004.

[22] Miyamoto Y, Sono T. Lessons from Peer Support Among Individuals with Mental Health Difficulties: A Review of the Literature. Clin Pract Epidemiol Ment Health 2012;8:22. https://doi.org/10.2174/1745017901208010022.

[23] Johnson S, Lamb D, Marston L, Osborn D, Mason O, Henderson C, et al. Peer-supported self-management for people discharged from a mental health crisis team: a randomised controlled trial. The Lancet 2018;392:409–18. https://doi.org/10.1016/S0140-6736(18)31470-3.

[24] Marshall Z, Dechman MK, Minichiello A, Alcock L, Harris GE. Peering into the literature: A systematic review of the roles of people who inject drugs in harm reduction initiatives. Drug Alcohol Depend 2015;151:1–14. https://doi.org/10.1016/J.DRUGALCDEP.2015.03.002.

[25] National Institute for Health and Care Excellence. Integrated health and social care for people experiencing homelessness 2022.

[26] Miler JA, Carver H, Foster R, Parkes T. Provision of peer support at the intersection of homelessness and problem substance use services: A systematic “state of the art” review. BMC Public Health 2020;20:1–18. https://doi.org/10.1186/S12889-020-8407-4/TABLES/4.

[27] Keats H, Maguire N, Johnson R, Cockersell P. Psychologically informed services for homeless people: Good Practice Guide 2012.

[28] Williamson E. Psychology in hostels briefing: Leaders in psychologically informed environments and trauma informed care. 2021.

[29] Phipps C. “Living here has changed me”: Resident and staff perceptions of Psychologically Informed Environments for homeless people. Thesis. 2016.

[30] Williamson E, Taylor K. Minding the margins: An innovation to integrate psychology in a homeless hostel environment. vol. 265, 2015, p. 33–7.

[31] Cockersell P. PIEs five years on. Mental Health and Social Inclusion 2016;20:221–30.

[32] Schneider C, Hobson CW, Shelton KH. ‘Grounding a PIE in the sky’: Laying empirical foundations for a psychologically informed environment (PIE) to enhance well-being and practice in a homeless organisation. Health Soc Care Community 2022;30:e657–67. https://doi.org/10.1111/HSC.13435.

[33] Ojo-Fati O, Joseph AM, Ig-Izevbekhai J, Thomas JL, Everson-Rose SA, Pratt R, et al. Practical issues regarding implementing a randomized clinical trial in a homeless population: Strategies and lessons learned. Trials 2017;18:1–10. https://doi.org/10.1186/S13063-017-2046-9/FIGURES/1.

[34] Simpson A, Flood C, Rowe J, Quigley J, Henry S, Hall C, et al. Results of a pilot randomised controlled trial to measure the clinical and cost effectiveness of peer support in increasing hope and quality of life in mental health patients discharged from hospital in the UK. BMC Psychiatry 2014;14:1–14. https://doi.org/10.1186/1471-244X-14-30/FIGURES/5.

[35] Parkes T, Matheson C, Carver H, Budd J, Liddell D, Wallace J, et al. Supporting Harm Reduction through Peer Support (SHARPS): Testing the feasibility and acceptability of a peer-delivered, relational intervention for people with problem substance use who are

 © University of Stirling

homeless, to improve health outcomes, quality of life and social functioning and reduce harms: Study protocol. Pilot Feasibility Stud 2019;5:1–15. https://doi.org/10.1186/S40814-019-0447-0/FIGURES/3.

[36] Foster R, Carver H, Wallace J, Dunedin A, Burridge S, Foley P, et al. “PPI? That sounds like Payment Protection Insurance”: Reflections and learning from a substance use and homelessness study Experts by Experience group. Res Involv Engagem 2021;7:1–10. https://doi.org/10.1186/S40900-021-00324-8/PEER-REVIEW.

[37] Parkes T, Matheson C, Carver H, Foster R, Budd J, Liddell D, et al. A peer-delivered intervention to reduce harm and improve the well-being of homeless people with problem substance use: the SHARPS feasibility mixed-methods study. Health Technol Assess 2022;26:1. https://doi.org/10.3310/WVVL4786.

[38] Homeless Link. The unhealthy state of homelessness: Health audit results 2014.

[39] Stone B, Dowling S, Cameron A. Cognitive impairment and homelessness: A scoping review. Health Soc Care Community 2019;27:e125–42. https://doi.org/10.1111/HSC.12682.

[40] Finch TL, Girling M, May CR, Mair FS, Murray E, Treweek S, et al. Improving the normalization of complex interventions: Part 2 - Validation of the NoMAD instrument for assessing implementation work based on normalization process theory (NPT). BMC Med Res Methodol 2018;18:1–13. https://doi.org/10.1186/S12874-018-0591-X/TABLES/9.

[41] National Institute for Health and Care Research. Payment guidance for researchers and professionals 2023.

[42] Carter BR, Hood K. Balance algorithm for cluster randomized trials. BMC Med Res Methodol 2008;8:1–8. https://doi.org/10.1186/1471-2288-8-65/TABLES/4.

[43] Bell ML, Rabe BA. The mixed model for repeated measures for cluster randomized trials: A simulation study investigating bias and type i error with missing continuous data. Trials 2020;21:1–10. https://doi.org/10.1186/S13063-020-4114-9/TABLES/6.

[44] Leyrat C, Morgan KE, Leurent B, Kahan BC. Cluster randomized trials with a small number of clusters: which analyses should be used? Int J Epidemiol 2018;47:321–31. https://doi.org/10.1093/IJE/DYX169.

[45] Fiero MH, Hsu CH, Bell ML. A pattern-mixture model approach for handling missing continuous outcome data in longitudinal cluster randomized trials. Stat Med 2017;36:4094–105. https://doi.org/10.1002/SIM.7418.

[46] The EuroQol Group. EuroQol - a new facility for the measurement of health-related quality of life. Health Policy (New York) 1990;16:199–208. https://doi.org/10.1016/0168-8510(90)90421-9.

[47] Al-Janabi H, Flynn TN, Coast J. Development of a self-report measure of capability wellbeing for adults: The ICECAP-A. Quality of Life Research 2012;21:167–76. https://doi.org/10.1007/S11136-011-9927-2/FIGURES/3.

[48] May CR, Albers B, Bracher M, Finch TL, Gilbert A, Girling M, et al. Translational framework for implementation evaluation and research: a normalisation process theory coding manual for qualitative research and instrument development. Implementation Science 2022;17:1–15. https://doi.org/10.1186/S13012-022-01191-X/FIGURES/1.

[49] Penchansky R, Thomas J. The concept of access: definition and relationship to consumer satisfaction. Med Care 1981;19:127–40.

[50] Barker S, Bishop F, Bodley Scott E, Stopa L, Maguire N. Developing a model of change mechanisms within Intentional Unidirectional Peer Support (IUPS). . Eur J Homelessness 2020;14:161–91.

 © University of Stirling
